# Photobiomodulation‑Engineered Extracellular Vesicles Enhance Neural Differentiation via UFL1‑Mediated UFMylation in Spinal Cord Injury

**DOI:** 10.1002/advs.77237

**Published:** 2026-08-18

**Authors:** Yunxiao Fang, Zuomeng Wu, Ruocheng Guo, Cancan Wang, Yue Qin, Yixiang Dong, Aoyun Li, Ao Liu, Chongyu Jia, Tianyu Han, Peiwen Song, Lei Chen, Cailiang Shen

**Affiliations:** ^1^ Department of Orthopedics (Spinal Surgery) The First Affiliated Hospital of Anhui Medical University Hefei People's Republic of China; ^2^ Laboratory of Spinal and Spinal Cord Injury Regeneration and Repair The First Affiliated Hospital of Anhui Medical University Hefei People's Republic of China; ^3^ Department of Orthopedics (Spinal Surgery) The People's Hospital of Bozhou Bozhou People's Republic of China; ^4^ School of Materials Science and Engineering Hefei University of Technology Hefei People's Republic of China; ^5^ Intelligent Manufacturing Institute of Hefei University of Technology Hefei People's Republic of China; ^6^ Anhui Province Research Center for the Clinical Application of Digital Medical Technology The First Affiliated Hospital of Anhui Medical University Hefei Anhui People's Republic of China

**Keywords:** engineered extracellular vesicles, neurodifferentiation, photobiomodulation, spinal cord injury, UFMylation

## Abstract

Spinal cord injury (SCI) often results in permanent motor dysfunction, and no approved therapy effectively promotes nerve regeneration. We show that engineered extracellular vesicles (EVs) from photobiomodulation (PBM)‐treated microglia, delivering UFL1 (the sole E3 ligase of the UFMylation system), significantly enhance neural repair after SCI. PBM (850 nm, 2.0 J/cm^2^) promoted microglial M2 polarization and suppressed M1. We isolated PBM‐EVs and identified UFL1 as the key effector via proteomic analysis. Mechanistically, UFL1 competes with MDM2 for p53 binding, inhibiting p53 ubiquitination and degradation, thus stabilizing p53 and activating its signaling. In vitro, PBM‐EV‐delivered UFL1 protected neural stem cells from inflammation‐induced apoptosis and promoted neuronal differentiation; these effects were markedly attenuated by UFL1 knockdown or p53 inhibition. In a rat SCI model, PBM‐EVs delivering UFL1 significantly improved the local immune microenvironment, promoted M2 microglial polarization, reduced glial scar formation, and enhanced axonal regeneration and hindlimb motor recovery. Pharmacological p53 inhibition with PFT‐α or UFL1 knockdown in donor EVs substantially diminished these therapeutic effects in vivo, confirming the central role of the UFL1/p53 axis. Overall, PBM‐EVs deliver UFL1 to activate p53 signaling, synergistically regulating immunity and neural differentiation to promote functional recovery, highlighting the UFL1/p53 axis as a therapeutic target for SCI.

## Graphical abstract

Photobiomodulated microglia release engineered extracellular vesicles that deliver UFL1 to the injured spinal cord. UFL1 competitively binds p53 with MDM2, inhibiting p53 ubiquitination and stabilizing p53 signaling. This dual mechanism simultaneously promotes anti‐inflammatory microglial polarization and directs neural stem cell differentiation toward neurons, ultimately enhancing functional recovery after spinal cord injury.

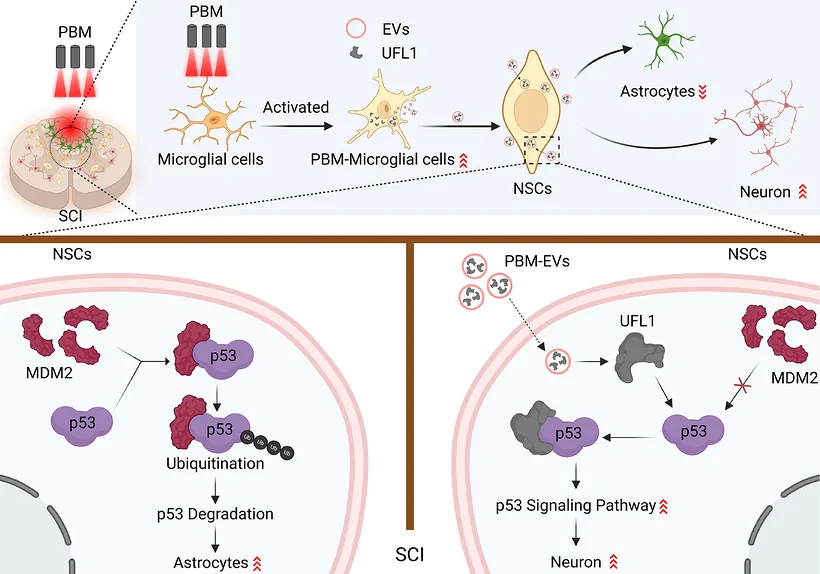

## 1 Introduction

Spinal cord injury (SCI) is a severe central nervous system trauma that often results in permanent loss of sensory and motor function below the injury level, imposing a substantial burden on patients, their families, and society [1, 2]. Globally, hundreds of thousands of new SCI cases are reported annually. However, clinical interventions for SCI remain limited, and strategies for effectively promoting nerve regeneration and functional reconstruction are not available [3, 4]. The pathophysiology of SCI involves primary mechanical injury followed by a secondary cascade of reactions, in which key events, including imbalance of the inflammatory microenvironment, neuronal and glial cell death, glial scar formation, and obstruction to axonal regeneration, collectively hinder the recovery of neural function [2, 5, 6].

In recent years, directing the differentiation of endogenous neural stem cells (NSCs) toward the neuronal lineage to replace damaged neurons has emerged as a prominent strategy in SCI repair. However, the injury site exhibits persistent inflammation and an acidic microenvironment, which severely impair NSC survival and bias their differentiation toward astrocytes, exacerbating glial scar formation and precluding functional reconstruction [5, 7]. Therefore, simultaneously improving the immune microenvironment and directing NSC neuronal differentiation represents a key challenge in SCI treatment.

Photobiomodulation (PBM), a non‐invasive physical therapy in which low‐intensity laser or LED light is used to regulate cellular function, has been shown to exert multiple biological effects, including anti‐inflammatory, pro‐survival, and pro‐regenerative activities [8, 9]. Photon absorption by cytochrome c oxidase within the mitochondrial respiratory chain enhances electron transport, elevating mitochondrial membrane potential, ATP production, and downstream anti‐inflammatory signaling. Under specific parameters, PBM promotes microglial polarization from the pro‐inflammatory M1 to the anti‐inflammatory M2 phenotype, ameliorating the neuroinflammatory microenvironment [10, 11]. However, lack of cellular specificity limits the ability of PBM to directly target deep‐layer NSCs or precisely control their differentiation fate.

Extracellular vesicles (EVs) are nanoscale membranous carriers that transfer proteins, mRNAs, and miRNAs between cells. Their low immunogenicity, high biocompatibility, and capacity to cross biological barriers have made them attractive therapeutic vectors for SCI [12, 13]. However, the efficacy of natural EVs is limited by their endogenous cargo repertoire. Engineered EVs, modified through donor cell preconditioning or direct loading, can enrich specific functional molecules and have shown promise in SCI repair. Recently, engineered EVs loaded with specific functional molecules, such as miR‐219, brain‐derived neurotrophic factor, or anti‐inflammatory factors, have been demonstrated to promote oligodendrocyte differentiation, enhance neuronal survival, and modulate the immune microenvironment, and thereby exerting multi‐level repair effects [14, 15, 16, 17]. These findings underscore the considerable potential of engineered EVs in SCI repair. However, given the fact that the pathophysiology of SCI involves interconnected events, including inflammation, apoptosis, glial scar formation, and axonal regeneration blockade, intervention with a single functional molecule often yields suboptimal outcomes. Thus, identifying key multi‐target effector molecules and developing multifunctional engineered EVs that confer immunomodulatory, neuroprotective, and pro‐differentiation effects is a critical research direction.

p53, canonically regarded as a tumor suppressor governing apoptosis and cell‐cycle arrest, has recently been implicated in stem cell fate determination: p53 deficiency promotes astrocytic differentiation of NSCs at the expense of neurogenesis, while p53 stabilization enhances neuronal commitment. However, the role of p53 in NSC fate decisions within the post‐SCI inflammatory microenvironment has not been explored. In this study, we investigated the therapeutic potential of photobiomodulation‐engineered extracellular vesicles (PBM‐EVs) in SCI. Using proteomics, we identified UFL1 as a key effector molecule within these vesicles. Mechanistically, PBM‐EVs activated the p53 signaling pathway via UFL1 delivery, which in turn modulated microglial polarization, protected NSCs from inflammation, and promoted neuronal differentiation. These findings position the UFL1/p53 axis as a promising target for SCI repair.

## 2 Materials and Methods

### 2.1 Isolation and Culture of Primary Microglia

Primary microglia were isolated from the cerebral cortex of newborn rats. The meninges and blood vessels were removed under sterile conditions and minced with scissors. The minced tissue was digested with 0.25% trypsin (Gibco, USA) at 37°C for 15 min. After termination of digestion, the digested tissue was mechanically pipetted into a single‐cell suspension, filtered through a 70 µm strainer, and centrifuged. The cell pellet, thus obtained, was resuspended in DMEM/F12 (HyClone, USA) supplemented with 10% fetal bovine serum (FBS, Gibco, USA) and 1% penicillin‐streptomycin, and the suspension was seeded into poly‐L‐lysine‐coated 75 cm^2^ culture flasks. After 9–12 days of culture, microglia were detached by shaking the flasks at 180 rpm for 1 h at 37°C. Floating cells were collected, centrifuged, and resuspended in DMEM/F12 containing 10% FBS and 20 ng/mL macrophage colony‐stimulating factor (M‐CSF, PeproTech, USA) and seeded into culture dishes for further culture.

### 2.2 PBM Treatment

Microglia were randomly assigned to different groups. Control cells were cultured without any intervention. Experimental groups were treated with lipopolysaccharide (LPS) or irradiated with a light‐emitting diode (LED) array for PBM. All procedures were conducted at a controlled ambient temperature of 23°C ± 2°C. To minimize interference from ambient light, cells were maintained under complete darkness or dim light except during PBM irradiation. Light source parameters are listed in Table 1, with measurements taken at a distance of 1 cm from the output port. The order of PBM irradiation across energy density groups and the assignment of culture dishes to treatment conditions were randomized using a random number table. For each independent experiment, the treatment sequence was re‐randomized.

**TABLE 1:** Dosimetric parameters for photobiomodulation.

Wavelength (nm)	850
**Work mode**	Continuous wave (CW)
Light source type	LED array
Irradiance at culture dish position without lid (mW/cm^2^)	∼5.8
Culture dish lid transmittance (%)	∼87.9
**Irradiance at cellular level (mW/cm^2^)**	∼**5.1**
Spot area (cm^2^)	20
**Energy density range tested (J/cm^2^)**	**0.5, 1.0, 2.0, 4.0**

### 2.3 Cell Transfection

For UFL1 knockdown, small interfering RNAs (siRNAs) targeting UFL1 and corresponding negative controls (GenePharma, Shanghai, China) were transfected into primary microglia using Lipofectamine 2000 reagent (Invitrogen, USA) according to the manufacturer's instructions. The validated siRNA target sequences were: sense, 5′‐GGAAGUUGUCAGAGCAUUAdTdT‐3′; antisense, 5′‐UAAUGCUCUGACAACUUCCdTdT‐3′. A non‑targeting scrambled siRNA (sense: 5′‐UUCUCCGAACGUGUCACGUdTdT‐3′; antisense: 5′‐ACGUGACACGUUCGGAGAAdTdT‐3′) was used as a negative control. Transfection was performed with 50 nM siRNA in Opti‑MEM reduced serum medium (Gibco). After 6 h, the medium was replaced with fresh DMEM/F12 containing 10% FBS. Cells were harvested 48 h post‑transfection, and knockdown efficiency was verified by quantitative real‑time PCR (qRT‑PCR).

### 2.4 Isolation, Purification, Identification, and Tracing of Engineered EVs

Engineered EVs were isolated from microglia subjected to PBM as described previously, with some modifications [18]. Briefly, after 24 h of PBM, the culture medium was collected and subjected to gradient centrifugation at 4 °C to remove cell debris. The supernatant was centrifuged at 100 000 × g for 90 min at 4°C using a Type 70 Ti rotor (Beckman Coulter, USA). The resulting crude EV pellet was washed by resuspension in 10 mL ice‐cold PBS and re‐centrifugation at 100 000 × g for 90 min at 4°C to remove co‐isolated protein aggregates and non‐vesicular components. The washed EV pellet was resuspended in 100 µL of sterile PBS and stored at −80°C until use. The particle size and morphology of EVs were analyzed via Nanoparticle tracking analysis (NTA) and transmission electron microscopy (TEM). Western blotting was performed to detect EV‐positive markers CD63, CD9, and TSG101 (1:1000, Abcam, UK) and the negative marker Calnexin (1:1000, Abcam, UK).

For tracing, EVs were labeled with PKH‐26 (Sigma, Germany) according to the manufacturer's instructions. Briefly, 4 µL of PKH‐26 was diluted in 100 µL of Diluent C and mixed with suspended EVs. After incubation at a controlled ambient temperature of 23°C ± 2°C for 10 min, the EVs were pelleted via centrifugation at 100 000 × g for 90 min at 4°C, washed twice with DMEM/F12 to remove the free dye, and immediately added to primary NSC cultures or used for tail vein injection in SD rats after injury.

### 2.5 Isolation and Culture Treatment of Primary NSCs

Primary NSCs were isolated from the subventricular zone of embryonic day 14–16 (E14–E16) fetal rats as previously described [19]. Briefly, the tissue was trypsinized at 37°C for 15 min, and cells were suspended in NSC culture medium containing DMEM/F12, 20 ng/mL EGF (Gibco, USA), 2% B27 (Gibco, USA), and 10 ng/mL bFGF (Gibco, USA). The cells were seeded at a density of 1 × 10^6^/mL in culture flasks and cultured, with the medium changed every three days. After 7 days of suspension culture, neurospheres were dissociated into single cells with trypsin and seeded into NSC differentiation medium (DMEM/F12, 2% B27, and 1% antibiotic solution). After 3 days of differentiation, NSCs were randomly divided into groups. Control cells were maintained in normal‐pH medium, whereas experimental group cells were treated with LPS to simulate the post‐SCI pathological microenvironment, with or without various EV types. After 24 h, immunofluorescence staining was performed.

### 2.6 Spinal Cord Injury Model Construction and Treatment

Animal experiments were approved by the Experimental Animal Ethics Committee of Anhui Medical University (approval No. LLSC 20242471). Female Sprague‐Dawley (SD) rats (8–10 weeks old, approximately 220 g) were obtained from the Laboratory Animal Center of Anhui Medical University (Hefei, China). Female rats were selected for this study to avoid potential confounding effects of aggressive behavior and fighting among male rats after spinal cord injury, which can compromise injury uniformity and animal welfare. Moreover, the SCI field has historically predominantly utilized female rodents for basic science research, allowing direct comparison with previous findings. Prior to surgery, rats were weighed and assigned to experimental groups using a computer‑generated block randomization sequence (block size = 3; GraphPad QuickCalcs, GraphPad Software, USA). Allocation was performed by an independent investigator not involved in surgery or outcome assessment. The allocated group was concealed in sequentially numbered opaque envelopes, opened only after SCI induction and confirmation of complete bilateral compression, which ensured that the surgeon was blinded to group allocation during the procedure. Spinal cord injury (SCI) was induced at the T9–T10 level using a standard operating procedure established by our group [20]. Briefly, rats were anesthetized with pentobarbital sodium (35 mg/kg, intraperitoneal injection), the T9–T10 spinal cord segment was exposed, and a complete bilateral compression injury was applied for 5 s using Dumont microforceps (tip width 0.5 mm). After surgery, broad‐spectrum antibiotics were administered twice daily, and manual bladder massage was performed to assist urination. Animals were housed under specific pathogen‐free conditions at 22°C–24°C and 40%–60% relative humidity, with a 12 h light/dark cycle and free access to standard rodent feed and water.

Rats were randomly assigned to six groups: sham, SCI, SCI + Control‐EVs, SCI + PBM‐EVs, SCI + siUFL1‐P‐EVs, and SCI + PBM‐EVs + p53 inhibitor. In the SCI group, animals underwent the same surgical procedure without further intervention. For treatment groups, EVs (100 µg total protein in 200 µL sterile PBS) were administered via tail vein injection immediately after surgery and on days 1, 3, and 7 post‐injury.

Group sizes were determined by a priori power analysis (G*Power 3.1) based on pilot BBB score data (effect size f = 0.65, α = 0.05, power = 0.80, one‐way ANOVA with 6 groups), yielding n = 8 per group. To accommodate anticipated attrition (estimated 10%–15% mortality), two additional animals were enrolled per injury group (total enrolled: 10 per injury group, 8 for sham). All animals surviving to day 28 with complete data were included in the final analysis. See Table S1 for the full animal flow.

### 2.7 Pharmacological Treatments

In vitro. PFT‐α (Pifithrin‐α hydrobromide, KKL Med, China) was dissolved in DMSO to a stock concentration of 50 mM and used at a final concentration of 10 µM in NSC culture medium (final DMSO concentration < 0.1%). For experiments involving PFT‐α co‐treatment, NSCs were pre‐incubated with PFT‐α for 2 h before EV addition, and the inhibitor was maintained throughout the culture period. Nutlin‐3a (KKL Med, China) was dissolved in DMSO and used at a final concentration of 5 µM under identical conditions. An equivalent volume of DMSO was added to control cultures.

In vivo. PFT‐α was freshly dissolved in DMSO (50 mg/mL stock) and diluted in sterile saline to a final concentration of 5 mg/mL (10% DMSO in saline). Rats in the SCI + PBM‐EVs + PFT‐α group received daily intraperitoneal injections of PFT‐α at 5 mg/kg body weight for 7 consecutive days, beginning 30 min prior to the first EV injection on the day of SCI. Vehicle control animals received equivalent volumes of 10% DMSO in saline.

### 2.8 Behavioral Assessment

Hindlimb motor function was evaluated using the Basso, Beattie, and Bresnahan (BBB) open‐field test and the inclined plane test. Locomotor recovery was assessed on days 1, 3, 7, 14, 21, and 28 post‐injury by two independent observers blinded to group allocation. Hindlimb motor coordination was assessed in a custom‐built clear acrylic swimming tank (120 cm × 30 cm × 40 cm) filled with tap water maintained at 25°C ± 1°C to a depth of 25 cm. Rats were gently placed at one end of the tank and allowed to swim freely toward a visible escape platform at the opposite end. Lateral and ventral video recordings were captured simultaneously using two synchronized high‐speed cameras. Videos were independently scored by two observers blinded to group allocation using the Louisville Swimming Scale (LSS; 0–14 scale), which evaluates hindlimb movement frequency, alternation pattern, body position, and forelimb dependency. Scores from the two observers were averaged; inter‐rater reliability was assessed by intraclass correlation coefficient (ICC > 0.90). Three consecutive swim trials were averaged per animal per time point. Gait was assessed at 4 weeks post‐injury. Forepaws were dipped in non‐toxic red ink and hindpaws in non‐toxic blue ink. Rats walked spontaneously across a paper‐lined runway (100 cm × 10 cm × 15 cm) toward a darkened goal box. Stride length and toe spread were measured from ≥6 consecutive, clearly defined steps per trial using ImageJ. Three trials per animal were averaged.

### 2.9 Electrophysiological Assessment

Electrophysiological assessment was performed at week 4 post‐injury to evaluate recovery of nerve conduction. For motor‐evoked potential (MEP) recording, the stimulating electrode was placed on the scalp surface 2.5 mm rostral to the anterior edge of the upper eyelid and 2.5 mm lateral to the midline, at a depth of 1 mm. The recording electrode was inserted into the gastrocnemius muscle belly, and the ground electrode was attached to the back skin in an interference‐free area. All electrodes were connected to a biological signal acquisition system for simultaneous data collection and analysis.

### 2.10 Immunofluorescence Staining

For immunofluorescence staining, animals were transcardially perfused with cold phosphate‐buffered saline (PBS) followed by 4% paraformaldehyde (PFA) for tissue fixation. Spinal cord tissues were harvested, post‐fixed in 4% PFA at 4°C for 24 h, and sectioned longitudinally at 4 µm thickness using a Leica RM2135 microtome (Leica, Germany), with sections centered at the injury site. For each staining batch, sections from all experimental groups were processed simultaneously, and the slide identity was masked by an independent investigator. The order of image acquisition was randomized across groups using a random number generator to avoid time‐of‐day or photobleaching confounds.

Sections were blocked with 10% bovine serum albumin (BSA) to prevent non‐specific binding, then incubated overnight at 4°C with primary antibodies: mouse anti‐Tuj1 (1:200, Abcam, UK), rabbit anti‐GFAP (1:1000, Abcam, UK), rabbit anti‐Iba1 (1:200, Abcam, UK), mouse anti‐iNOS (1:200, Abcam, UK), rabbit anti‐Arg1 (1:200, Abcam, UK), mouse anti‐UFL1 (1:200, Abcam, UK), and rabbit anti‐p53 (1:200, Abcam, UK). After washing, sections were incubated with fluorescently labeled secondary antibodies (1:50, Elabscience, China) for 1 h at room temperature, followed by nuclear counterstaining with 4′,6‐diamidino‐2‐phenylindole (DAPI). Images were acquired using a fluorescence microscope (AxioVert A1, Imager A2, Zeiss, Germany).

Quantification of immunofluorescence in spinal cord tissue. For each animal, 4–6 longitudinal sections (4 µm thickness), spaced 40 µm apart and centered on the lesion epicenter, were analyzed per staining panel. Regions of interest (ROIs) spanning 500 µm immediately rostral and caudal to the lesion border (Z1 and Z2) and at the lesion center (Z3) were defined. Within each ROI, 3–4 non‐overlapping fields were captured at 20× magnification using identical acquisition settings (exposure time, gain, lamp intensity) across all groups within each staining batch. Fluorescence intensity thresholds were determined by the Otsu automatic thresholding algorithm applied to sham‐group images and were held constant across all experimental groups within the same batch. For cell counting, DAPI^+^ nuclei within the ROI were automatically segmented (ImageJ/Fiji, NIH), and the percentage of marker‐positive cells (iNOS^+^, Arg1^+^, Tuj1^+^, GFAP^+^, NeuN^+^, NF^+^ area) among total DAPI^+^ nuclei or Iba1^+^ cells was calculated. For the triple‐stained tissue sections, iNOS^+^/Iba1^+^ and Arg1^+^/Iba1^+^ proportions were scored within the same fields. NF fluorescence intensity was quantified as the mean integrated density along a line profile (width = 100 µm) spanning 1 mm centered on the lesion epicenter.

For cell immunofluorescence, cells were fixed with 4% PFA for 30 min, permeabilized with 0.05% Triton X‐100, blocked with 10% BSA, and incubated with primary and secondary antibodies as described above.

All histological quantification was performed by an investigator blinded to group allocation. Specifically, group identities were replaced with numerical codes by an independent investigator prior to image acquisition and analysis. Codes were revealed only after all quantitative data for a given staining batch had been recorded. For immunofluorescence, identical acquisition parameters (exposure time, gain, lamp intensity) were applied across all groups within each batch. Fluorescence thresholds were determined using the Otsu algorithm on sham group images and applied uniformly. Cell counting was performed using automated segmentation (ImageJ/Fiji) with minimal manual adjustment, and all adjustments were applied uniformly across all images in the batch.

### 2.11 HE Staining and Nissl Staining

For histological analysis, spinal cord sections were stained with hematoxylin and eosin (HE) according to the reagent instructions, and images were captured under an optical microscope. Nissl staining was performed following the manufacturer's protocol: sections were incubated in Nissl staining solution at 37°C for 10 min, briefly rinsed with double‐distilled water for 5 s, and dehydrated in 95% ethanol for 5 s to terminate the reaction. Images were acquired for assessment of neuronal structural integrity and distribution.

To evaluate systemic toxicity, tissue samples from the heart, liver, spleen, lung, and kidney were collected on day 14 post‐injury for HE staining. Bladder tissue was collected on day 28 for HE staining, and blood samples were collected to assess liver and kidney function markers. These analyses were used to evaluate the safety of the intervention.

### 2.12 Cell Counting Kit‐8 (CCK‐8) Assay

Primary NSCs were seeded into 96‐well plates at 5 × 10^3^ cells per well in 100 µL of culture medium and incubated for 24 h. After the designated treatments, 10 µL of CCK‐8 reagent (C0037, Beyotime Biotechnology) was added to each well, followed by further incubation for 1 h. Optical density (OD) was measured at 450 nm using a microplate reader (LUX, Thermo Fisher Scientific, USA) to assess cell viability.

### 2.13 Apoptosis Detection (TUNEL Staining and Flow Cytometry)

Primary NSCs were seeded at 1 × 10^5^ cells per well and subjected to the designated treatments. Apoptosis was assessed by two complementary methods: TUNEL staining and flow cytometry. For TUNEL staining, cells were processed using a TUNEL detection kit (Beyotime Biotechnology, China) according to the manufacturer's instructions, and the apoptosis rate was quantified. For flow cytometry analysis, NSCs were harvested, washed twice with cold PBS, and resuspended in 100 µL of 1× Annexin V Binding Buffer (Beyotime Biotechnology, China). Cells were then incubated with 5 µL of Annexin V‐FITC and 5 µL of propidium iodide (PI) (Beyotime Biotechnology, China) for 15 min at room temperature in the dark. After adding 400 µL of 1× Binding Buffer, samples were immediately analyzed on a CytoFLEX SRT Flow Cytometer Sorter (Beckmancoulter, USA), with a minimum of 10,000 events recorded per sample. Quadrant gates were defined using unstained, Annexin V‐FITC single‐stained, and PI single‐stained controls as follows: Q1‐LL (Annexin V^−^/PI^−^, viable cells; lower‐left), Q1‐LR (Annexin V^+^/PI^−^, early apoptotic cells; lower‐right), Q1‐UR (Annexin V^+^/PI^+^, late apoptotic cells; upper‐right), and Q1‐UL (Annexin V^−^/PI^+^, necrotic cells; upper‐left). Total apoptotic rate was calculated as Q1‐LR + Q1‐UR. Data were analyzed using FlowJo v10.8 (TreeStar, USA).

### 2.14 Reactive Oxygen Species (ROS) Detection

For ROS detection in NSCs, cells were seeded at 1 × 10^5^ cells per well and subjected to the designated treatments. Cells were washed twice with cold PBS, incubated with diluted 2′,7′‐dichlorodihydrofluorescein diacetate (DCFH‐DA) (Beyotime, S0033S, Shanghai, China) at 37°C for 20 min, washed three times with serum‐free medium, and examined under a fluorescence microscope (DM6B, Leica, Wetzlar, Germany) to detect ROS production.

For tissue ROS detection, sections were pre‐warmed to restore physiological conditions, stained with DHE working solution at 37°C for 30 min, washed three times with PBS, counterstained with DAPI, and imaged under the same microscope.

### 2.15 Quantitative Reverse Transcription Polymerase Chain Reaction (qRT‐PCR)

Total RNA was extracted from tissues, cells, and EVs using TRIzol reagent (Gibco, USA). Complementary DNA (cDNA) was synthesized with the Superscript III RT reaction mixture (Invitrogen). Quantitative PCR was performed on a 7900 Fast Real‐Time PCR System (Applied Biosystems) using SYBR Green PCR Master Mix. Glyceraldehyde 3‐phosphate dehydrogenase (GAPDH) was used as an internal reference for normalization, and relative expression was calculated using the 2^−ΔΔCt^ method. Primer sequences were as follows:

Tuj1: forward 5′‐AAGAATTCAGCAGATGCTGGCCATTCAGAGTA‐3′, reverse 5′‐AATCTAGATAAACTGCTCGGAGATGCGCTTGA‐3′
GFAP: forward 5′‐AAGGATCCACAGACTTTCTCCAACCTCCAG‐3′, reverse 5′‐AAGAATTCCCTTCTGACACGGATTTGGT‐3′
UFL1: forward 5′‐AAGAATTCATGGCGGAGGCGGAGCGG‐3′, reverse 5′‐AATCTAGATCAGGCATACTCGTACACCATCTG‐3′
GAPDH: forward 5′‐AAGAATTCAAGGGCTCATGACCACAGTC‐3′, reverse 5′‐AATCTAGAGTGAGCTTCCCATTCAGCTC‐3′

### 2.16 Enzyme‐Linked Immunosorbent Assay (ELISA)

Spinal cord tissue samples, 5 mm segments centered on the lesion epicenter, were homogenized in ice‐cold RIPA lysis buffer (Beyotime, China) containing a protease inhibitor cocktail using a tissue homogenizer (60 Hz, 30 s × 3 cycles). Homogenates were centrifuged at 12 000 × g for 15 min at 4°C. Supernatant total protein concentration was determined by the BCA Protein Assay Kit (Beyotime, China). Concentrations of TNF‐α, IL‐1β, IL‐6, IL‐4, and IL‐10 were measured using rat‐specific ELISA kits (Beyotime, China) according to the manufacturer's instructions. Cytokine concentrations were normalized to total protein content and expressed as pg cytokine per mg total protein. All samples were assayed in duplicate, and the mean intra‐assay coefficient of variation was < 10%.

### 2.17 Western Blotting

Cells or tissues were lysed in RIPA buffer containing protease inhibitors. Protein concentrations were determined using the BCA method. Equal amounts of protein were separated by SDS‐PAGE, transferred to PVDF membranes, and blocked with 5% non‐fat milk. Membranes were incubated overnight at 4°C with primary antibodies, followed by incubation with HRP‐conjugated secondary antibodies (1:15 000) for 2 h at room temperature. Bands were visualized using an ECL luminescence reagent and the GelDoc XR system (Bio‐Rad, USA). Protein expression levels were quantified using ImageJ software by normalizing target band intensity to that of control bands. For Western blot densitometry, band intensities were quantified using ImageJ by an investigator blinded to group identity. Group labels on membrane images were replaced with numerical codes before quantification.

### 2.18 Proteomics Sequencing Analysis of Engineered EVs

Engineered EVs (PBM‐EVs) were isolated from the culture supernatant of PBM‐treated microglia via differential ultracentrifugation, with EVs from untreated microglia serving as controls. EV preparations meeting quality criteria based on particle size, morphology, and marker expression were subjected to proteomic analysis. Following protein extraction, reduction, alkylation, and tryptic digestion, peptides were purified, vacuum‐dried, and separated on an EASY‐nLC 1200 system coupled to a mass spectrometer. Raw data acquisition was performed at the Metabolomics Laboratory of OE Biotech (Shanghai, China) using a timsTOF MS platform (Bruker, Germany) operating in data‐independent acquisition mode. Subsequent analyses included differential protein screening, functional enrichment, and protein–protein interaction network construction. Three biological replicates were analyzed per group.

### 2.19 Co‐Immunoprecipitation (Co‐IP)

Plasmids encoding N‐terminally Flag‐tagged UFL1 and N‐terminally Myc‐tagged p53 were constructed and transfected into primary NSCs. After 48 h, the cells were lysed in IP lysis buffer, and the lysate was incubated with anti‐Flag affinity gel overnight at 4°C. The samples were washed, separated via SDS‐PAGE, and subjected to western blotting with anti‐Flag, anti‐Myc, and anti‐MDM2 antibodies to detect protein interactions. IgG and empty vector controls were included.

### 2.20 Ubiquitination Assay

To assess p53 ubiquitination, primary NSCs were co‐transfected with plasmids expressing His‐tagged ubiquitin (His‐Ub) and p53. After 48 h, the cells were treated with the proteasome inhibitor MG132 (10 µM) for 6 h to enrich ubiquitinated proteins. The cells were lysed in denaturing lysis buffer, and lysates were incubated with the corresponding antibody overnight at 4°C, followed by incubation with Protein A/G agarose beads for 4 h. Eluted products were separated via SDS‐PAGE and subjected to western blotting with an anti‐p53 antibody to detect ubiquitinated p53, which appeared as a ladder‐like pattern of high‐molecular‐weight bands. Empty vector‐transfected and MG132‐untreated groups served as controls. To examine whether MDM2 promotes p53 ubiquitination, an MDM2 expression plasmid was co‐transfected, and changes in p53 ubiquitination were assessed.

### 2.21 Statistical Analysis

Sample size determination. Group sizes for animal experiments were determined by a priori power analysis (G*Power 3.1.9.7) using pilot BBB score data from our laboratory. For a one‐way ANOVA design with 6 groups, an effect size f = 0.65 (derived from the observed difference of 3.7 points in 28‐day BBB scores between PBM‐EV and Control‐EV groups in the pilot study), α = 0.05, and power = 0.80, the required sample size was n = 8 per group. To accommodate anticipated attrition (estimated 10%–15% mortality and exclusion), two additional animals were enrolled per injury group (total enrolled: n = 10 per injury group; sham: n = 8).

Animal enrollment, exclusion, and attrition. Pre‐specified exclusion criteria were: (i) death within 72 h of surgery; (ii) autophagia or self‐mutilation requiring early euthanasia; (iii) incomplete injury, defined as a BBB score > 3 on day 1 post‐injury. Animals meeting any exclusion criterion were removed from the study and not replaced. The number of animals enrolled, excluded, and included in the final analysis for each group is reported in Table S1. No animals were excluded after day 7 post‐injury.

Randomization. Animals were assigned to experimental groups using a computer‐generated block randomization sequence (block size = 3) generated by an independent investigator. Allocation was concealed in sequentially numbered opaque envelopes opened after SCI induction and confirmation of injury completeness. For in vitro experiments, treatment allocations were determined by a random number table and re‐randomized for each independent experiment. For histological analyses, slide identities were masked and imaging order was randomized across groups.

Blinding. Blinding was maintained at all stages: (i) the surgeon was blinded to group allocation during SCI induction; (ii) all behavioral assessments were scored by two independent observers blinded to group identity; (iii) histological quantification, Western blot densitometry, and flow cytometry gating were performed with group identities masked by numerical codes; (iv) statistical analysis was performed on de‐identified datasets. Group codes were revealed only after all analyses were complete.

Normality testing and statistical methods. Data are presented as mean ± standard deviation (SD). Normality was assessed using the Shapiro–Wilk test. For normally distributed data, two‐group comparisons used unpaired two‐tailed Student's *t*‐test and multiple‐group comparisons used one‐way ANOVA with Tukey's HSD post‐hoc test. For non‐normally distributed data, the Mann–Whitney *U* test (two groups) or Kruskal–Wallis test with Dunn's correction (multiple groups) was applied. Longitudinal behavioral data (BBB scores) were analyzed by repeated‐measures two‐way ANOVA (group × time) with Geisser–Greenhouse correction. A *p*‐value < 0.05 was considered statistically significant. All analyses were performed using GraphPad Prism 8 and SPSS 26.0.

Reproducibility. All in vitro experiments were performed in at least three independent biological replicates, each with technical duplicates or triplicates as specified in individual figure legends. An independent biological replicate was defined as an experiment performed on a separate day using a freshly thawed cell aliquot or independently prepared EV batch. All attempts at replication were successful. An ARRIVE 2.0 Essential 10 checklist is provided as File S1.

## 3 Results

### 3.1 PBM Promotes Microglial M2 Polarization and Inhibits M1 Polarization

A PBM irradiation device was developed using an 850 nm LED array emitting continuous‐wave (CW) light (Figure 1A). Light penetration through the culture dish lid was evaluated with a spectrometer. The incident irradiance was 5.8 mW/cm^2^ at the culture dish plane without the lid. With the culture dish lid in place, approximately 87.9% of incident light was transmitted, yielding an irradiance at the cellular level of 5.1 mW/cm^2^ (Figure 1B,C).

**FIGURE 1:**
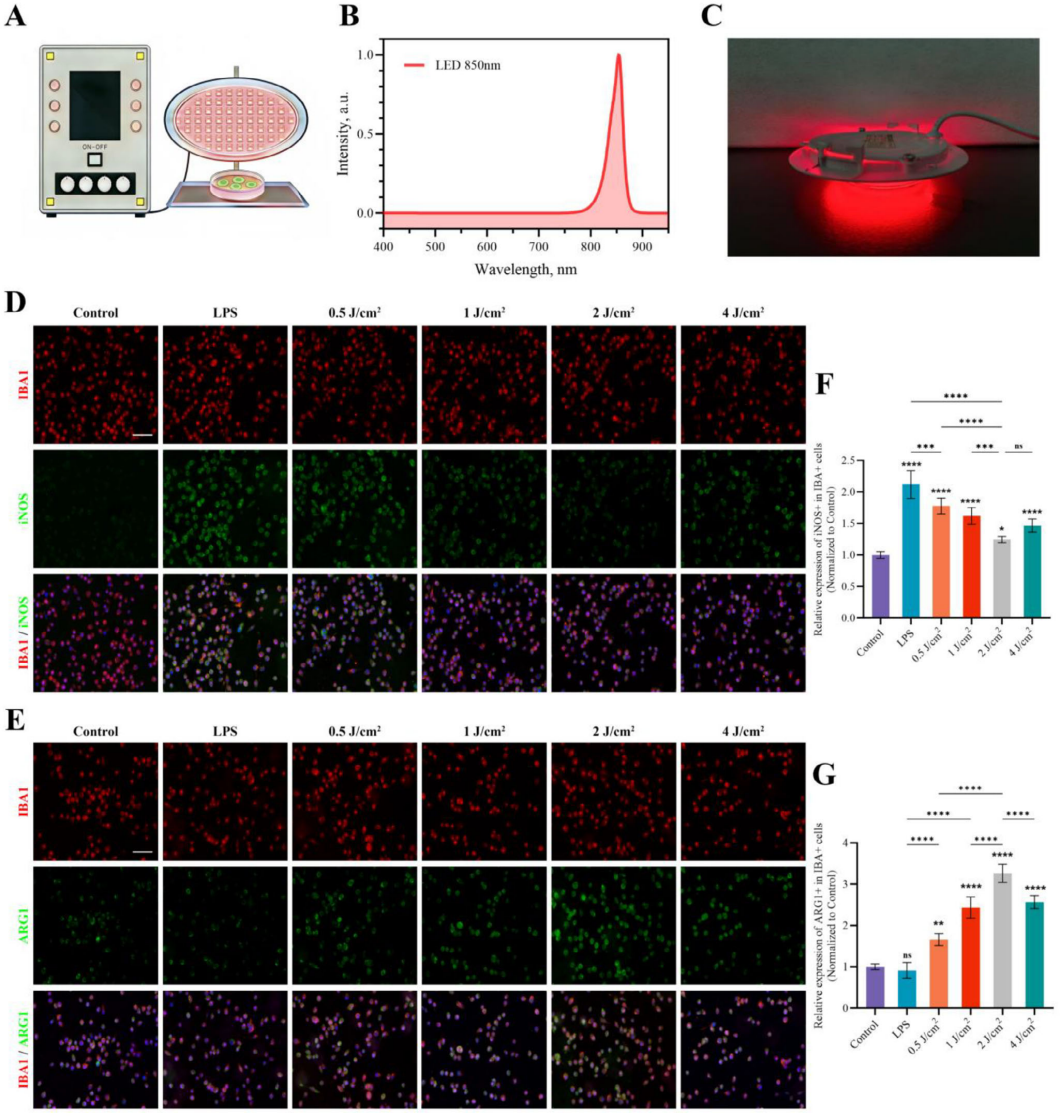
Characterization of the PBM device and its effect on microglial polarization. (A)Schematic diagram of the PBM irradiation device configured with an 850 nm LED array. (B) Emission spectrum of the LED array showing the peak wavelength at 850 nm. (C) Actual image of a Petri dish irradiated by the PBM irradiation device. (D, E) Representative immunofluorescence images of iNOS (D) and Arg1 (E) in microglia after PBM irradiation at different energy densities (0.5, 1.0, 2.0, and 4.0 J/cm^2^) or LPS treatment (scale bars, 50 µm). (F, G) Quantitative analysis of iNOS^+^ in Iba1^+^ cells (F) and Arg1^+^ in Iba1^+^ cells (G) cell proportions under each condition. n = 3 independent biological replicates (independent cell cultures performed on separate days); ≥3 fields quantified per replicate. All data are presented as mean ± SD. Statistical analysis: one‐way ANOVA with Tukey's post‐hoc test. ns, *p* > 0.05; ^*^ *p* < 0.05; ^**^ *p* < 0.01; ^***^ *p* < 0.001; ^****^ *p* < 0.0001.

To determine optimal PBM parameters, microglia were irradiated at 850 nm with energy densities of 0.5, 1.0, 2.0, and 4.0 J/cm^2^. iNOS and Arg1, as marker proteins reflecting M1 and M2 polarization, respectively, can indicate the polarization and functional status of microglia. Immunofluorescence staining (Figure 1D–G) showed that PBM increased the proportion of Arg1‐positive cells and decreased that of iNOS‐positive cells relative to that in controls. LPS treatment significantly increased iNOS expression, indicating M1 activation. Among the tested conditions, microglia reached peak Arg1 expression after exposure at an energy density of 2.0 J/cm^2^ and subsequent culture for 24 h. These in vitro dose‐escalation experiments served as a preliminary screen to identify the optimal PBM energy density for subsequent EV production. Definitive M1/M2 polarization was subsequently assessed by triple immunofluorescence in spinal cord tissue. Collectively, these results indicated that at 2.0 J/cm^2^, PBM effectively induced microglial polarization toward the anti‐inflammatory M2 phenotype.

### 3.2 Preparation and Characterization of PBM‐EVs

To investigate whether M2 microglia induced by PBM regulate neural differentiation after SCI via EV secretion, EVs were isolated from microglia culture supernatant using differential ultracentrifugation with a PBS wash step, as described in §2.4 (Figure 2A). NTA revealed that the modal particle size of Control‐EVs was 211 nm (D10–D90: 130–310 nm) and that of PBM‐EVs was 181 nm (D10–D90: 120–250 nm) (Figure 2B). The modest size reduction in PBM‐EVs may reflect alterations in membrane lipid composition, protein cargo density, or membrane curvature induced by PBM‐mediated metabolic reprogramming of donor microglia, consistent with previous reports of activity‐dependent changes in EV biophysical properties. Both preparations showed size distributions within the small EV range, though the broader distribution of Control‐EVs indicates more heterogeneous vesicle populations. TEM revealed typical cup‐shaped or spherical vesicles, and both Control‐EVs and PBM‐EVs exhibited diameters within the normal range (Figure 2C). Western blot analysis confirmed the presence of EV markers CD9, CD63, and TSG101 in both Control‐EV and PBM‐EV preparations, and importantly, the endoplasmic reticulum marker Calnexin was detected in donor microglial cell lysate but was absent from both EV preparations (Figure 2D). Collectively, these data are consistent with the MISEV2023‐recommended characterization of small EV‐enriched preparations.

**FIGURE 2:**
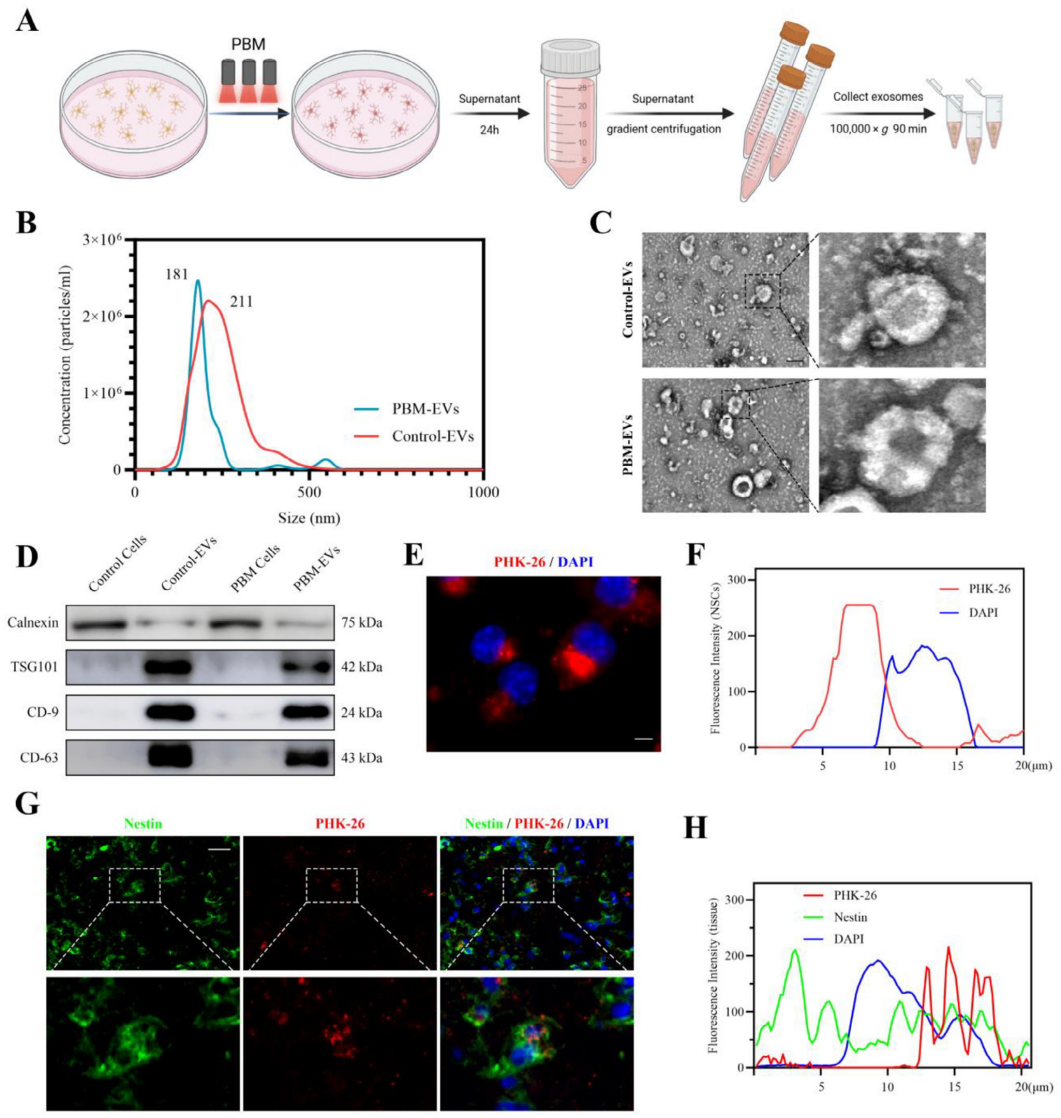
Isolation and characterization of PBM‐engineered EVs and their uptake by NSCs. (A) Schematic diagram of the EV isolation procedure using differential ultracentrifugation. (B) Nanoparticle tracking analysis (NTA) showing the size distribution of Control‐EVs and PBM‐EVs. (C) Representative transmission electron microscopy (TEM) images of Control‐EVs and PBM‐EVs (scale bars, 200 nm). (D) Western blot analysis of EV markers in Control‐EVs and PBM‐EVs. n = 3 independent experiments; representative blot shown. Densitometry normalized to loading control. (E, F) Uptake of PKH26‐labeled EVs by primary NSCs. (E; scale bars, 20 µm). n = 3 independent biological replicates. (F) Quantification of fluorescence intensity along the line scan, demonstrating effective cellular internalization of EVs. (G) Uptake of PKH26‐labeled EVs in the spinal cord of SCI rats 24 h after tail vein injection, demonstrating that systemically administered EVs can reach the injured spinal cord parenchyma. (scale bars, 50 µm). n = 5 animals per group. (H) Fluorescence intensity line scan across the region of interest, confirming EV accumulation at the injury site. All data are presented as mean ± SD. Statistical analysis: one‐way ANOVA with Tukey's post‐hoc test. ns, *p* > 0.05; ^*^ *p* < 0.05; ^**^ *p* < 0.01; ^***^ *p* < 0.001; ^****^ *p* < 0.0001.

To assess internalization by primary NSCs, PKH26‐labeled EVs were co‐incubated with NSCs for 4 h. Confocal microscopy revealed red fluorescence distributed in the cytoplasm, indicating the effective uptake of EVs (Figure 2E,F). Similarly, following tail vein injection of PKH26‐labeled EVs in SCI rats, red fluorescence was detected in the spinal cord parenchyma at the injury site (Figure 2G,H), indicating that systemically administered EVs can cross the compromised blood–spinal cord barrier and accumulate at the lesion. PBM treatment did not alter the morphology or marker expression of EVs.

### 3.3 PBM‐EVs Modulate the Immune Microenvironment After SCI

Suppressing inflammation in the early phase after SCI can attenuate secondary injury and create a favorable microenvironment for nerve repair. To evaluate the regulatory effect of PBM‐EVs on the immune microenvironment, a rat SCI model was established, and the animals were treated with PBS, Control‐EVs, or PBM‐EVs (see the experimental timeline in Figure 3A). On day 7 post‐surgery, injured tissue was collected, and concentrations of pro‐inflammatory and anti‐inflammatory cytokines at the injury center were measured using enzyme‐linked immunosorbent assay (ELISA). Compared with those in the SCI group, pro‐inflammatory cytokines TNF‐α, IL‐1β, and IL‐6 were significantly downregulated, whereas anti‐inflammatory cytokines IL‐4 and IL‐10 were significantly upregulated, with the most pronounced effects observed in the PBM‐EV group (Figure 3B–F).

**FIGURE 3:**
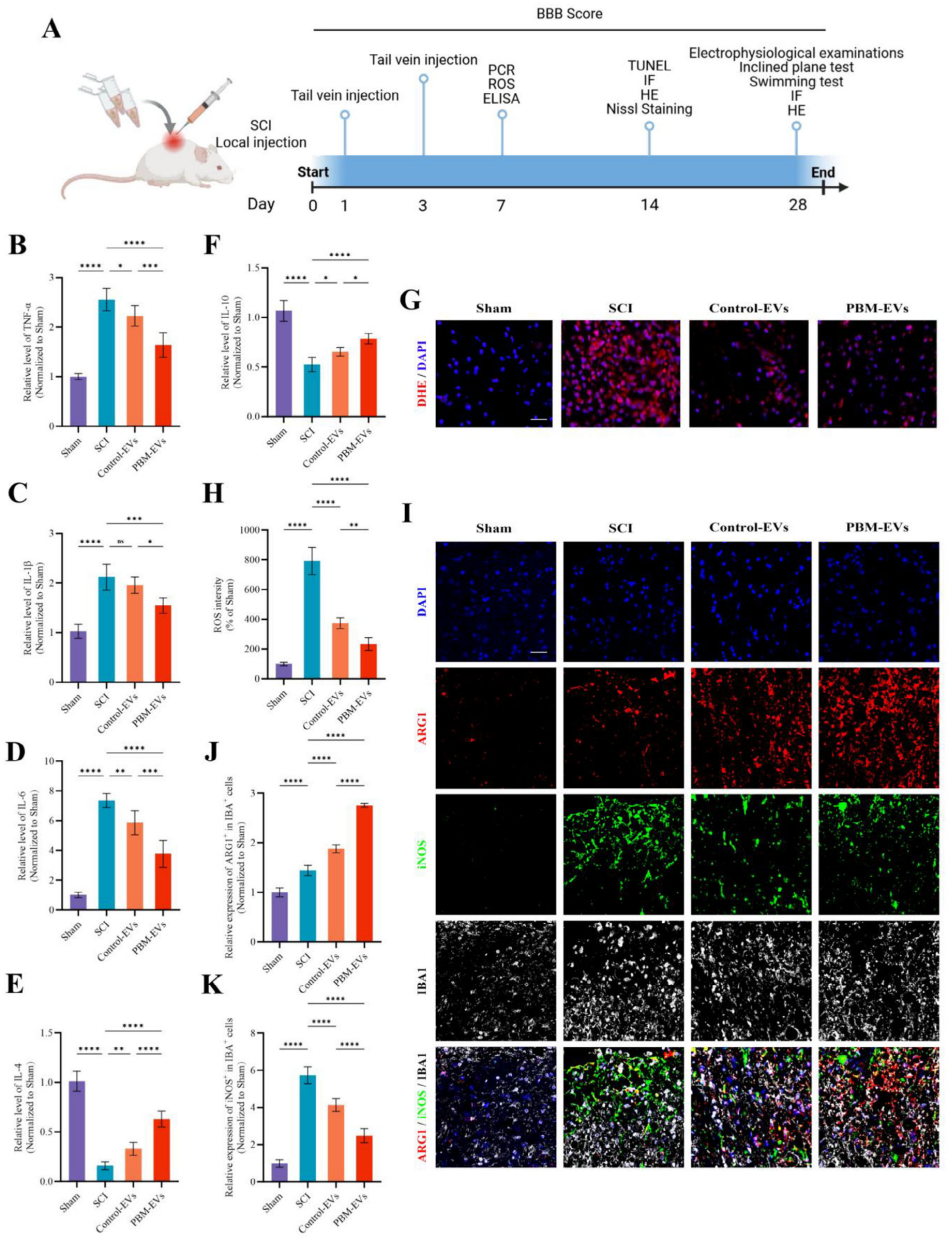
PBM‐EVs modulate the post‐SCI immune microenvironment and reduce oxidative stress. (A) Experimental timeline of the rat spinal cord clamp model. (B–F) ELISA quantification of pro‐inflammatory cytokines TNF‐α (B), IL‐1β (C), IL‐6 (D) and anti‐inflammatory cytokines IL‐4 (E), IL‐10 (F) in spinal cord tissue at 7 days post‐injury. (G) Dihydroethidium (DHE) staining for superoxide anion (ROS) levels at the injury site (scale bars, 50 µm). (H) Quantification of DHE fluorescence intensity. (I) Representative images of triple immunofluorescence staining for iNOS, Arg1, and Iba1 on spinal cord sections (scale bars, 50 µm). (J) Quantification of iNOS^+^/Iba1^+^ cell proportion. (K) Quantification of Arg1^+^/Iba1^+^ cell proportion. n = 5 animals per group for ELISA and histological analyses. All data are presented as mean ± SD. Statistical analysis: one‐way ANOVA with Tukey's post‐hoc test. ns, *p* > 0.05; ^*^ *p* < 0.05; ^**^ *p* < 0.01; ^***^ *p* < 0.001; ^****^ *p* < 0.0001.

Oxidative stress was assessed by measuring the levels of superoxide anion using dihydroethidium (DHE) staining (Figure 3G,H). ROS production was elevated after SCI compared with that in the sham group, indicating mitochondrial dysfunction. EV treatment reduced oxidative stress, with PBM‐EVs showing the strongest inhibitory effect. Immunofluorescence triple staining for iNOS, Arg1, and Iba1 on spinal cord sections revealed that in the PBM‐EV group, the proportion of Arg1‐positive microglia among Iba1‐positive cells was significantly increased, whereas that of iNOS‐positive microglia was decreased, yielding an elevated M2/M1 ratio within the same microscopic fields (Figure 3I–K). These results indicate that PBM‐EVs effectively modulate the post‐SCI immune microenvironment and promote microglial polarization toward the anti‐inflammatory M2 phenotype.

### 3.4 PBM‐EVs Promote Neural Regeneration in SCI

On day 14 post‐injury, TUNEL staining revealed obvious cell apoptosis, which was significantly reduced by EV treatment, with PBM‐EVs showing the strongest anti‐apoptotic effect (Figure S1A,B). Nissl staining showed that in the SCI group, Nissl bodies at the injury center were faint and neurons were sparse, whereas in the PBM‐EV group, more neurons with intact morphology and abundant Nissl bodies were observed in the peri‐injury area (Figure S1C,D). NeuN immunofluorescence staining corroborated these findings: rats treated with PBM‐EVs exhibited a greater number of NeuN‐positive neurons in the rostral region adjacent to the injury site compared with that in the Control‐EV group (Figure 4A,B), indicating a protective effect of PBM‐EVs on peri‐injury neurons.

**FIGURE 4:**
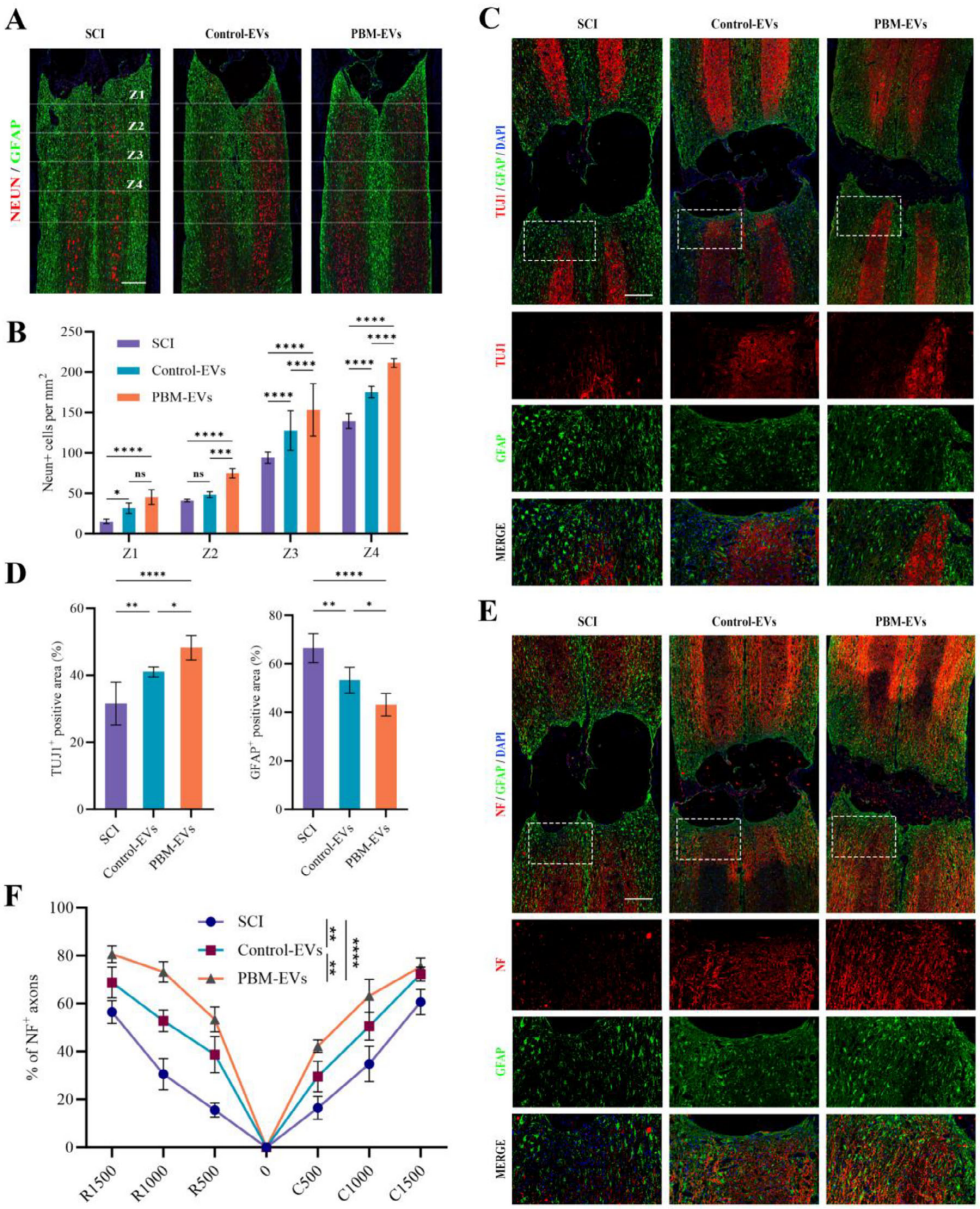
PBM‐EVs promote neuronal survival, axonal regeneration, and reduce glial scar formation after SCI. (A) Representative immunofluorescence images of NeuN in the peri‑injury spinal cord on day 28 post‑injury (scale bars, 200 µm). (B) Quantification of NeuN‑positive neurons in the Z1–Z3 regions. (C) Double immunofluorescence staining of Tuj1 and GFAP in the injury site on day 28 (scale bars, 200 µm). (D) Quantitative analysis showing that PBM‑EVs markedly increased the Tuj1‑positive area while decreasing the GFAP‑positive area compared with Control‑EVs. (E) Representative images of NF and GFAP co‑staining in sections of the injured spinal cord (scale bars, 200 µm). (F) Quantification of NF‑positive fluorescence intensity across the injury site presented as a line graph. n = 5 animals per group for histological quantification. All data are presented as mean ± SD. Statistical analysis: one‐way ANOVA with Tukey's post‐hoc test. ns, *p* > 0.05; ^*^ *p* < 0.05; ^**^ *p* < 0.01; ^***^ *p* < 0.001; ^****^ *p* < 0.0001.

On day 28 post‐injury, both EV treatments increased axonal formation at the injury center and reduced glial scar formation, with PBM‐EVs showing the most pronounced effect (Figure 4C,D). Axonal regeneration, assessed by examining NF‐positive neurofilaments, was significantly enhanced in the PBM‐EV group compared with that in the Control‐EV group (Figure 4E,F). Collectively, these results indicate that PBM‐EVs promote axonal regeneration, and neuronal survival after SCI, providing a structural basis for functional recovery.

### 3.5 PBM‐EVs Promote Functional Recovery in SCI

Behavioral recovery was assessed using the Basso, Beattie, and Bresnahan (BBB) open‐field test (Figure 5A). After SCI, rats exhibited complete hindlimb paralysis. Motor recovery in treatment groups became statistically significant at 2 weeks post‐surgery, and by 4 weeks, the PBM‐EV group showed greater improvement than the Control‐EV group. The inclined plane test yielded similar results at 4 weeks, with PBM‐EV‐treated rats exhibiting the largest inclined angle and best grip strength recovery (Figure 5B). Swimming tests further revealed significant improvement in hindlimb motor coordination by PBM‐EVs (Figure 5C,D).

**FIGURE 5:**
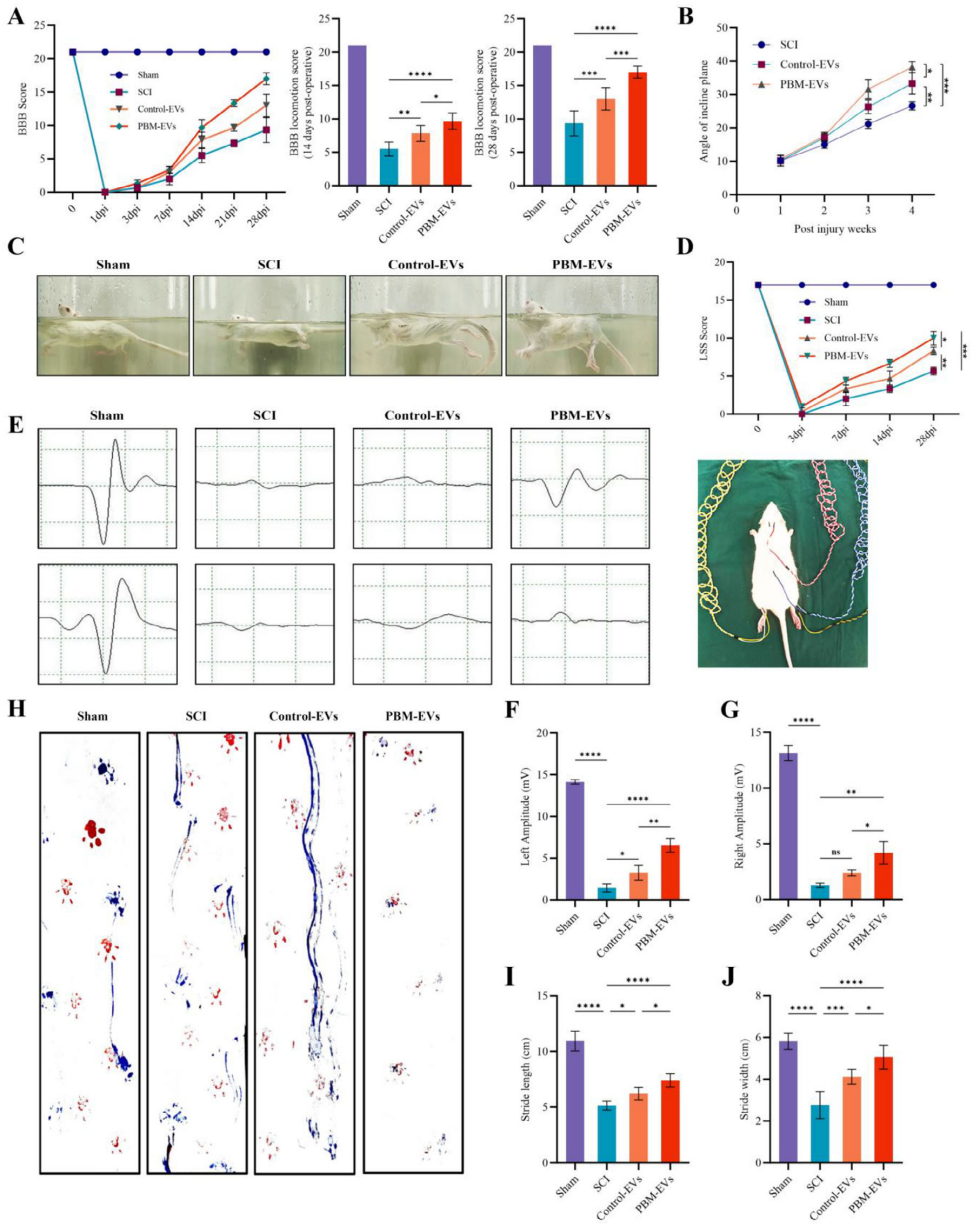
PBM‐EVs promote functional recovery after SCI. (A) Basso, Beattie, and Bresnahan (BBB) open‑field scores over 4 weeks post‑injury. (B) Inclined plane test at 4 weeks post‑injury. (C, D) Swimming test analysis of hindlimb motor coordination. (C) Swimming test assessment of motor coordination in rats at 4 weeks post‐injury. (D) LSS score analysis derived from the swimming test at multiple time points in rats. (E) MEP‐based neurophysiological assessment in rats at 4 weeks post‐injury. (F, G) Quantification of MEP amplitude in the left and right hindlimbs at 4 weeks post‑injury. (H–J) Footprint analysis at 4 weeks post‑injury. (H) Representative footprints of rats from each group. (I, J) Quantification of stride length and toe spacing. n = 8 animals per group for behavioral assessments. All data are presented as mean ± SD. Statistical analysis: one‐way ANOVA with Tukey's post‐hoc test. ns, *p* > 0.05; ^*^ *p* < 0.05; ^**^ *p* < 0.01; ^***^ *p* < 0.001; ^****^ *p* < 0.0001.

Neuroelectrophysiological assessment of motor‐evoked potential (MEP) was performed at 4 weeks post‐injury. Stimulation of the subcutaneous tissue over the head elicited gastrocnemius muscle signals (Figure 5E). MEP amplitude was significantly increased after EV treatment, indicating recovery of injured neural circuits, with the greatest increase observed in PBM‐EV‐treated rats (Figure 5F,G). Footprint analysis at 4 weeks showed clear footprints, regular gait, increased stride length, and improved toe spacing in the PBM‐EV group, whereas control rats exhibited footprint dragging and unstable gait (Figure 5H–J).

Histological analysis at 28 days post‐injury revealed cavity formation at the injury center due to neuronal loss. EV treatment significantly reduced the cavity area, with PBM‐EVs showing the strongest effect (Figure S2A,B). Bladder structure analysis indicated that PBM‐EVs promoted bladder remodeling and prevented bladder wall thinning after SCI (Figure S2C–E). Safety evaluation showed that organ structures (heart, liver, spleen, lung, and kidney) were intact and comparable to those of sham‐operated animals (Figure S2F). Collectively, these findings indicate that PBM‐EVs promote functional recovery after SCI without apparent toxicity.

### 3.6 PBM‐EVs Protect NSCs From Inflammatory Environments

To model the post‐SCI pathological microenvironment, NSCs were treated with LPS, as previously described [19]. After 24 h, LPS‐treated NSCs were incubated with different EVs. TUNEL staining revealed significantly increased apoptosis in the inflammatory model, which was reduced after EV treatment, with the most pronounced effect observed in the PBM‐EV group (Figure 6A,B). Flow cytometry confirmed these findings: the proportion of apoptotic cells was significantly lower in the PBM‐EV group than in the model group, whereas the Control‐EV group showed no significant difference (Figure 6C).

**FIGURE 6:**
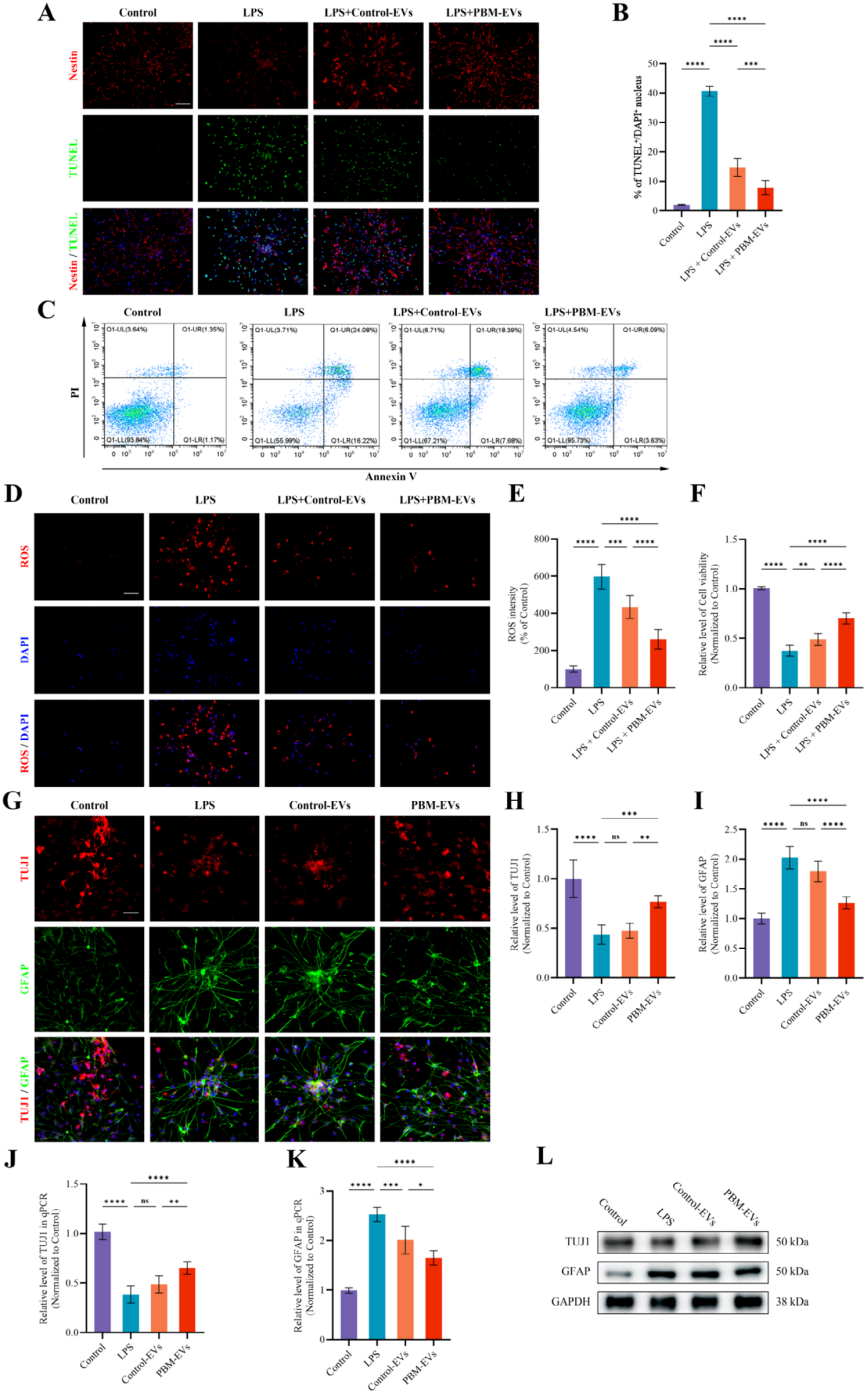
PBM‑EVs protect neural stem cells (NSCs) from inflammation‑induced apoptosis and oxidative stress, and promote NSC differentiation toward neurons while inhibiting astrocyte differentiation. (A) Representative immunofluorescence images of Nestin and TUNEL co‑staining in NSCs after 24 h of LPS treatment with or without EV incubation (scale bars: 50 µm). n = 3 independent biological replicates. (B) Quantification of TUNEL‑positive cells, expressed as the number of TUNEL‑positive nuclei per DAPI‑positive nuclei per field. (C) Flow cytometry analysis of NSC apoptosis using Annexin V‐FITC/PI double staining. Quadrants: Q1‐LL (viable), Q1‐LR (early apoptosis), Q1‐UR (late apoptosis), Q1‐UL (necrosis). Total apoptotic rate = Q1‐LR + Q1‐UR. n = 3 independent experiments; 10 000 events recorded per sample. (D) Representative images of reactive oxygen species (ROS) production detected by DCFH‑DA fluorescence (scale bars: 50 µm). n = 3 independent biological replicates. (E) Quantitative analysis of relative ROS fluorescence intensity. (F) CCK‑8 assay of NSC viability. n = 3 independent experiments with 6 technical replicates per condition. (G–I) Immunofluorescence analysis of NSC differentiation after 7 days in differentiation medium. n = 3 independent biological replicates. (G) Representative images of Tuj1 and GFAP co‑staining in NSCs treated with PBS, Control‑EVs, or PBM‑EVs under LPS‑induced inflammatory conditions (scale bars: 50 µm). (H) Quantification of Tuj1‑positive neurons. (I) Quantification of GFAP‑positive astrocytes. (J, K) qPCR analysis of NSC differentiation markers. n = 3 independent biological replicates, each assayed in triplicate. (J) Relative mRNA expression of Tuj1. (K) Relative mRNA expression of GFAP. (L) Western blot analysis of neuronal (Tuj1) and astrocytic (GFAP) proteins in differentiated NSCs. n = 3 independent experiments; representative blot shown. Densitometry normalized to the loading control. All data are presented as mean ± SD. Statistical analysis: one‐way ANOVA with Tukey's post‐hoc test. ns, *p* > 0.05; ^*^ *p* < 0.05; ^**^ *p* < 0.01; ^***^ *p* < 0.001; ^****^ *p* < 0.0001.

ROS production, measured using the DCFH‐DA probe, was elevated in the inflammatory model; PBM‐EVs effectively reduced the ROS levels, as indicated by decreased fluorescence intensity (Figure 6D,E). ELISA revealed that compared with the LPS‐treated group, EV treatment downregulated pro‐inflammatory cytokines (TNF‐α, IL‐1β, and IL‐6) and upregulated anti‐inflammatory cytokines (IL‐4 and IL‐10), with PBM‐EVs again showing the strongest effects (Figure S3A–E). CCK‐8 assays revealed that LPS‐induced reduction in NSC viability was ameliorated by EV treatment, particularly by PBM‐EVs (Figure 6F). Collectively, these results indicate that PBM‐EVs enhance NSC survival under inflammatory conditions.

### 3.7 PBM‐EVs Regulate NSCs Differentiation

To investigate the effect of EVs on NSC differentiation, primary NSCs were cultured as neurospheres for 7 days, dissociated into single cells, and seeded into differentiation medium. The cells were randomly divided into groups and subjected to LPS treatment with or without EVs (PBS, Control‐EVs, or PBM‐EVs). After 7 days of differentiation, EV treatment significantly altered the differentiation direction of NSCs, as evidenced by immunofluorescence staining. The PBM‐EV group exhibited a higher proportion of Tuj1‐positive neurons and a lower proportion of GFAP‐positive astrocytes compared with the Control‐EV group (Figure 6G–I). These findings were corroborated by qPCR results, which showed upregulation of the neuronal marker Tuj1 and downregulation of the astrocyte marker GFAP in the PBM‐EV group (Figure 6J,K). Western blot analysis confirmed similar changes in the expression of neural differentiation‐related proteins (Figure 6L). Collectively, PBM‐EVs promoted directional differentiation of NSCs toward neurons while reducing astrocyte differentiation.

### 3.8 PBM‐EVs Drive Microglial M2 Polarization by Delivering UFL1

To identify key effector molecules in PBM‐EVs, proteomic analysis was performed on Control‐EVs and PBM‐EVs (Figure 7A). The levels of 445 proteins were significantly increased and those of 952 proteins were decreased in PBM‐EVs compared with those in Control‐EVs (Figure 7B). Hierarchical clustering of differentially expressed proteins clearly distinguished the two groups (Figure 7C). GO enrichment analysis showed that upregulated proteins were primarily associated with biological processes, including rRNA processing, ribosomal small subunit biogenesis, and ribosomal large subunit biogenesis; cellular components, such as nucleolus, small‐subunit processome, and extracellular space; and molecular functions, including RNA helicase activity, RNA binding, and cohesin loader activity (Figure 7D). KEGG pathway analysis revealed significant enrichment in pathways including the p53 signaling pathway, ribosome biogenesis in eukaryotes, ECM–receptor interaction, ATP‐dependent chromatin remodeling, and N‐glycan biosynthesis (Figure 7E). Among the differentially expressed proteins, UFL1 abundance was markedly higher in PBM‐EVs than in Control‐EVs. Furthermore, UFL1 occupied a central node in the protein–protein interaction network, with potential interactions with proteins involved in neural differentiation and immune regulation (Figure 7F). High UFL1 expression in PBM‐EVs was independently confirmed via qRT‐PCR (Figure S4A). Western blot analysis of independent EV preparations confirmed that UFL1 protein abundance was significantly elevated in PBM‐EVs compared with Control‐EVs (Figure S4B), corroborating the proteomic and qRT‐PCR results.

**FIGURE 7:**
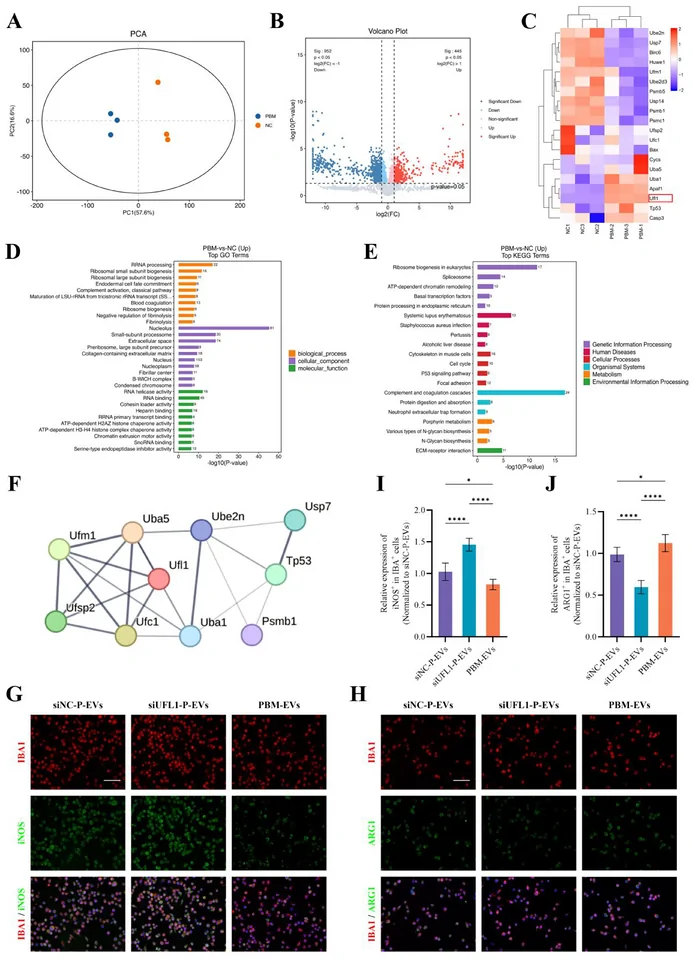
Proteomic analysis identifies UFL1 as a key effector molecule in PBM‐EVs, which drives microglial M2 polarization. (A) Principal component analysis (PCA) of protein expression profiles in Control‑EVs and PBM‑EVs (n = 3 per group), showing clear separation between the two groups with good within‑group consistency. (B) Volcano plot of differentially expressed proteins. (C) Hierarchical clustering heatmap of differentially expressed proteins, demonstrating distinct clustering between Control‑EV and PBM‑EV samples. (D) Gene Ontology (GO) enrichment analysis of upregulated proteins in PBM‑EVs, showing top terms in biological process (BP), cellular component (CC), and molecular function (MF). (E) Kyoto Encyclopedia of Genes and Genomes (KEGG) pathway enrichment analysis of differentially expressed proteins. (F) Protein–protein interaction (PPI) network analysis. (G–H) Representative immunofluorescence images of iNOS and Arg1 in LPS‑stimulated microglia after co‑incubation with siNC‐P‑EVs, siUFL1‐P‐EVs, or PBM‐EVs (scale bars, 50 µm). n = 3 independent biological replicates. (I) Quantification of iNOS‑positive cells. (J) Quantification of Arg1‑positive cells. All data are presented as mean ± SD. Statistical analysis: one‐way ANOVA with Tukey's post‐hoc test. ns, *p* > 0.05; ^*^ *p* < 0.05; ^**^ *p* < 0.01; ^***^ *p* < 0.001; ^****^ *p* < 0.0001.

UFL1, the only E3 ligase in the UFMylation system, has been implicated in immune regulation and cell differentiation [21, 22, 23]. To investigate its functional role in microglia, UFL1 was knocked down using siRNA, and EVs derived from these cells after the same PBM treatment (siUFL1‐P‐EVs) were prepared. UFL1 knockdown efficiency in donor microglia was confirmed by qRT‐PCR (Figure S4C). LPS‐stimulated rat primary microglia were co‐cultured with different EVs for 24 h. PBM‐EVs significantly increased the levels of the M2 marker Arg1 and decreased those of the M1 marker iNOS, whereas siUFL1‐P‐EVs did not show this effect (Figure 7G–J). This in vitro analysis served as an initial functional readout of UFL1‐dependent microglial polarization. Moreover, as evidenced from ELISA results, compared with PBM‐EVs, siUFL1‐P‐EVs treatment resulted in upregulation of pro‐inflammatory cytokines (TNF‐α, IL‐1β, and IL‐6) and downregulation of anti‐inflammatory cytokines (IL‐4 and IL‐10), with levels comparable to those in the LPS group (Figure S4D–H). Critically, the M1/M2 polarization shift was independently confirmed in spinal cord tissue by triple immunofluorescence staining for iNOS, Arg1, and Iba1 within the same microscopic fields, which demonstrated that PBM‐EVs significantly increased the Arg1^+^/Iba1^+^ proportion and decreased the iNOS^+^/Iba1^+^ proportion relative to both Control‐EVs and siUFL1‐P‐EVs (Figure S5A–C). Collectively, these results indicate that PBM‐EVs drive microglial M2 polarization through UFL1 delivery.

PBM‐EVs show a distinct molecular signature compared with conventionally polarized M2‐EVs. To determine whether the therapeutic cargo of PBM‐EVs reflects a general property of M2‐polarized microglia or a PBM‐specific effect, we prepared M2‐EVs from microglia treated with IL‐4 (20 ng/mL, 24 h) and compared their UFL1 content with PBM‐EVs. Immunofluorescence staining for Arg1/Iba1 and iNOS/Iba1 on donor microglia confirmed that both PBM and IL‐4 treatment effectively induced M2 polarization, with comparable increases in proportions of cells positive for both Arg1 and Iba1 and decreases in proportions of cells positive for both iNOS and Iba1 (Figure S6A–D). Western blot analysis revealed that UFL1 protein was significantly enriched in PBM‐EVs compared with M2‐EVs, indicating that PBM upregulates UFL1 beyond the level achieved by conventional M2 polarization alone (Figure S6E). These findings demonstrate that UFL1 enrichment is not a generic feature of M2‐polarized microglia but rather a PBM‐specific molecular trait, further supporting the qualitative distinction of PBM‐EVs from conventionally derived M2‐EVs.

### 3.9 PBM‐EVs Promote Neural Differentiation of NSCs and Functional Recovery by Transferring UFL1

To determine whether PBM‐EVs deliver UFL1 to NSCs, immunofluorescence staining was performed after co‐incubation. A robust UFL1 signal was observed in the cytoplasm and perinuclear region of NSCs treated with PBM‐EVs, whereas no such accumulation was detected in the Control‐EV group (Figure 8A,B). In vitro differentiation experiments showed that PBM‐EVs increased the proportion of Tuj1‐positive neurons and decreased the proportion of GFAP‐positive astrocytes. This effect was abrogated by UFL1 knockdown, as siUFL1‐P‐EV treatment resulted in Tuj1 and GFAP proportions comparable to the control levels (Figure 8C–E). Quantitative analysis confirmed that UFL1 is required for the pro‐neuronal differentiation effect of PBM‐EVs. In the SCI rat model, immunofluorescence double staining of peri‐injury tissue revealed that PBM‐EVs increased the proportion of Tuj1‐positive neurons and reduced the proliferation of GFAP‐positive astrocyte, whereas siUFL1‐P‐EVs had no significant effect (Figure 8F–H). However, as Tuj1 labels both pre‐existing and newly generated neurons, we performed EdU to directly assess whether PBM‑EVs promote de novo neurogenesis in the injured spinal cord. EdU (50 mg/kg, i.p.) was administered via intraperitoneal injection on day 7 post‑injury, and 24 h later the animals were processed for tissue staining. At that time, neurons that were double‑positive for EdU and NeuN were quantified in the peri‑lesion gray matter (Figure S7A,B). In the SCI group, newly generated neurons positive for both EdU and NeuN were detected at low levels, consistent with the limited spontaneous neurogenesis reported in the adult spinal cord after injury. Control‑EV treatment did not significantly alter this number. In contrast, PBM‑EV treatment significantly increased the density of EdU‑and‑NeuN‑positive neurons compared with the SCI group. This pro‑neurogenic effect was dependent on UFL1, as animals treated with siUFL1‑P‑EV exhibited densities of EdU‑and‑NeuN‑positive neurons comparable to those in the Control‑EV group and lower than those in the PBM‑EV group. These data provide evidence that PBM‑EVs promote the generation of new neurons from proliferating cells in the post‑SCI spinal cord via UFL1‑dependent mechanisms. Behavioral assessments using the BBB and LSS scales showed that recovery of motor function in the siUFL1‐P‐EV group was significantly impaired compared with that in the PBM‐EV group, and did not show any difference compared with the Control‐EVs group (Figure S7C,D). Collectively, these results indicate that PBM‐EVs promote NSC neuronal differentiation and functional recovery after SCI through UFL1 delivery.

**FIGURE 8:**
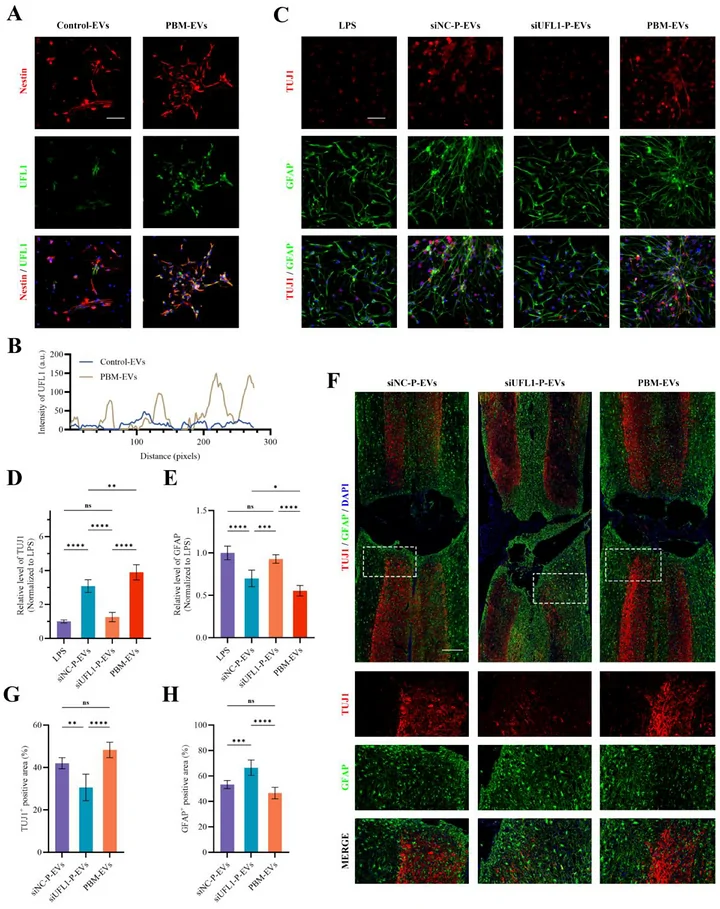
PBM‐EVs deliver UFL1 to NSCs and promote neuronal differentiation both in vitro and in vivo. (A, B)Immunofluorescence staining of UFL1 in NSCs after co‐incubation with Control‑EVs or PBM‑EVs for 4 h (scale bars, 20 µm). n = 3 independent biological replicates. (C) Representative images of Tuj1 and GFAP double‑immunofluorescence staining in NSCs treated with siNC‐P‐EVs, siUFL1‐P‐EVs, or PBM‐EVs, under LPS‐induced inflammatory conditions (scale bars, 50 µm). n = 3 independent biological replicates. (D) Quantification of Tuj1‐positive neurons. (E) Quantification of GFAP‑positive astrocytes. (F–H) In vivo analysis of NSC differentiation in the SCI rat model. n = 5 animals per group for histological analyses. (F) Representative immunofluorescence images of Tuj1 and GFAP in the peri‐injury spinal cord tissue from rats treated with siNC‐P‐EVs, siUFL1‐P‐EVs, or PBM‐EVs (scale bars, 100 µm). (G) Quantification of Tuj1‐positive area. (H) Quantification of GFAP‐positive area. n = 5 animals per group for histological analyses. All data are presented as mean ± SD. Statistical analysis: one‐way ANOVA with Tukey's post‐hoc test. ns, *p* > 0.05; ^*^ *p* < 0.05; ^**^ *p* < 0.01; ^***^ *p* < 0.001; ^****^ *p* < 0.0001.

### 3.10 UFL1‐Mediated UFMylation Competitively Inhibits MDM2‐Mediated p53 Ubiquitination

UFMylation stabilizes substrate proteins by antagonizing ubiquitination [24, 25]. Consistent with our proteomics data, GSEA showed significant enrichment of p53 signaling pathway‐related genes after PBM‐EV treatment (Figure S7E). To investigate the mechanism underlying the regulatory effect of UFL1 on p53 stability, we performed Co‐IP. Endogenous UFL1 and p53 were detected in reciprocal immunoprecipitates, indicating physical interaction (Figure 9A). This interaction was confirmed by co‐transfection of Flag‐tagged UFL1 and Myc‐tagged p53: Myc‐p53 co‐precipitated with anti‐Flag, but not with control IgG (Figure 9B,C). Knockdown of UFL1 (si‐UFL1) in microglia significantly reduced p53 protein levels, an effect that was largely reversed by the proteasome inhibitor MG132, suggesting that UFL1 maintains p53 stability by inhibiting proteasomal degradation (Figure 9D–F). Cycloheximide (CHX) chase experiments showed that the half‐life of p53 was shortened in si‐UFL1‐treated cells compared with that in control (si‐NC), indicating accelerated degradation (Figure 9G,H). In vivo ubiquitination assays revealed increased p53 ubiquitination upon UFL1 knockdown (Figure 9I,J), confirming the regulation of p53 stability by UFL1.

**FIGURE 9:**
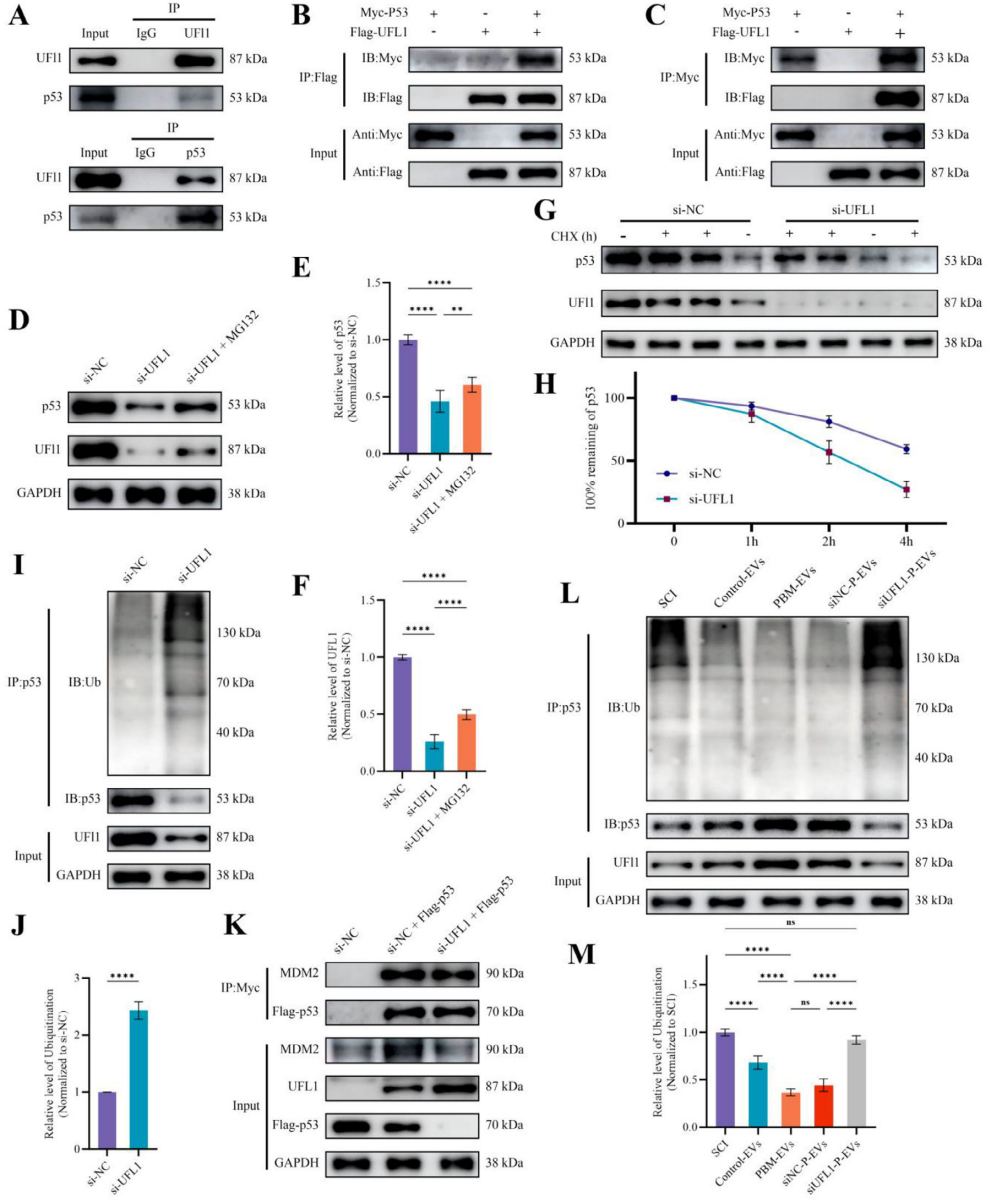
UFL1 stabilizes p53 by competitively inhibiting MDM2‑mediated ubiquitination. (A) Reciprocal co‑immunoprecipitation (Co‑IP) of endogenous UFL1 and p53 in microglia. (B, C) Co‑IP of exogenous Flag‑UFL1 and Myc‑p53. (B) Lysates from cells co‑transfected with Flag‑UFL1 and Myc‑p53 were immunoprecipitated with anti‑Flag antibody, followed by Western blotting for Myc‑p53. (C) Reciprocal Co‑IP using anti‑Myc antibody followed by anti‑Flag Western blotting. (D) Western blot analysis of p53 and UFL1 after UFL1 knockdown (si‑UFL1) with or without MG132 treatment (10 µM, 6 h). (E, F) Quantification of UFL1 and p53 protein levels from (D). (G) Cycloheximide (CHX) chase experiment. Cells were treated with si‑NC or si‑UFL1, followed by CHX (50 µg/mL) for the indicated times. (H) Quantification of p53 protein levels from (G). (I) In vivo ubiquitination assay for p53 in cells. (J) Quantification of p53 ubiquitination levels from (I). (K) Co‑IP of MDM2 with p53. Flag‑p53 was expressed in si‑NC or si‑UFL1 cells, and lysates were immunoprecipitated with anti‑Flag, followed by Western blotting for MDM2. (L) In vivo p53 ubiquitination assay in the SCI rat model. (M) Quantification of p53 ubiquitination levels in spinal cord tissues from (L). n = 3 independent experiments; representative blot shown. Densitometry normalized to loading control. All Data are presented as mean ± SD. ns, *p* > 0.05; ^*^ *p* < 0.05; ^**^ *p* < 0.01; ^***^ *p* < 0.001; ^****^ *p* < 0.0001.

p53 is continuously ubiquitinated and degraded by the 26S proteasome under normal conditions, a process primarily mediated by the E3 ubiquitin ligase MDM2 [26, 27, 28]. To explore the relationship between UFL1 and MDM2, MDM2 was co‐transfected, which further enhanced p53 ubiquitination in si‐UFL1 cells, suggesting competition between UFL1 and MDM2. Co‐IP using Flag‐p53 showed that MDM2–p53 binding was enhanced upon UFL1 knockdown (Figure 9K), indicating that UFL1 competes with MDM2 in binding with p53, thereby inhibiting MDM2‐mediated ubiquitination. In the SCI rat model, p53 ubiquitination was assessed on day 7 post‐surgery. Compared with the SCI group, the PBM‐EV group exhibited reduced p53 ubiquitination, whereas the siUFL1‐P‐EV group showed ubiquitination levels comparable to those in the Control‐EV group (Figure 9L,M). Collectively, these findings demonstrate that UFL1, as an E3 ligase in the UFMylation system, competes with MDM2 in binding to p53, inhibiting MDM2‐mediated ubiquitination and degradation of p53, thereby stabilizing it and facilitating downstream signaling.

### 3.11 UFL1‐Mediated PBM‐EVs Regulate NSC Differentiation via the p53 Signaling Pathway In Vitro and In Vivo

To investigate the downstream mechanism by which UFL1 in PBM‐EVs regulates neural stem cell differentiation, the involvement of the p53 signaling pathway was examined. Given that our previous experiments demonstrated that UFL1 stabilizes p53 protein by competitively inhibiting MDM2‐mediated p53 ubiquitination, we further validated the necessity of the p53 pathway in PBM‐EV‑induced neuronal differentiation. Immunofluorescence staining revealed significantly enhanced nuclear p53 fluorescence and its perinuclear co‑localization with UFL1 in PBM‑EV‑treated NSCs (Figure 10A–F). Figure 10G–I shows that under an inflammatory microenvironment induced by LPS, NSCs tended to differentiate into astrocytes with a low proportion of neurons. PBM‑EV treatment markedly increased the percentage of Tuj1‑positive neurons while decreasing GFAP‑positive astrocytes, indicating promotion of neuronal differentiation and suppression of glial differentiation. Co‑treatment with the p53‑specific inhibitor PFT‑α (10 µM) largely abolished these effects, bringing the neuronal proportion back to a level similar to that of the LPS group and increasing the astrocyte proportion accordingly, suggesting that p53 pathway activity is essential for the effects of PBM‑EVs. The p53 stabilizer Nutlin‑3a alone mimicked the pro‑neuronal differentiation effect of PBM‑EVs, significantly increasing the neuronal proportion. No additive effect was observed when Nutlin‑3a was combined with PBM‑EVs, indicating that they act through the same p53‑dependent pathway. To clarify the role of UFL1, NSCs treated with siUFL1‑P‑EVs failed to show enhanced neuronal differentiation, similar to control EVs. However, adding Nutlin‑3a to siUFL1‑P‑EV‑treated cells partially restored neuronal differentiation, with a Tuj1‑positive proportion clearly higher than that of the siUFL1‑P‑EVs alone group, though still not reaching the level of the PBM‑EVs group. Collectively, these results indicate that PBM‑EVs activate the p53 signaling pathway by delivering UFL1, thereby specifically promoting the differentiation of NSCs toward a neuronal lineage, and pharmacological stabilization of p53 can partially compensate for the loss of UFL1 function.

**FIGURE 10:**
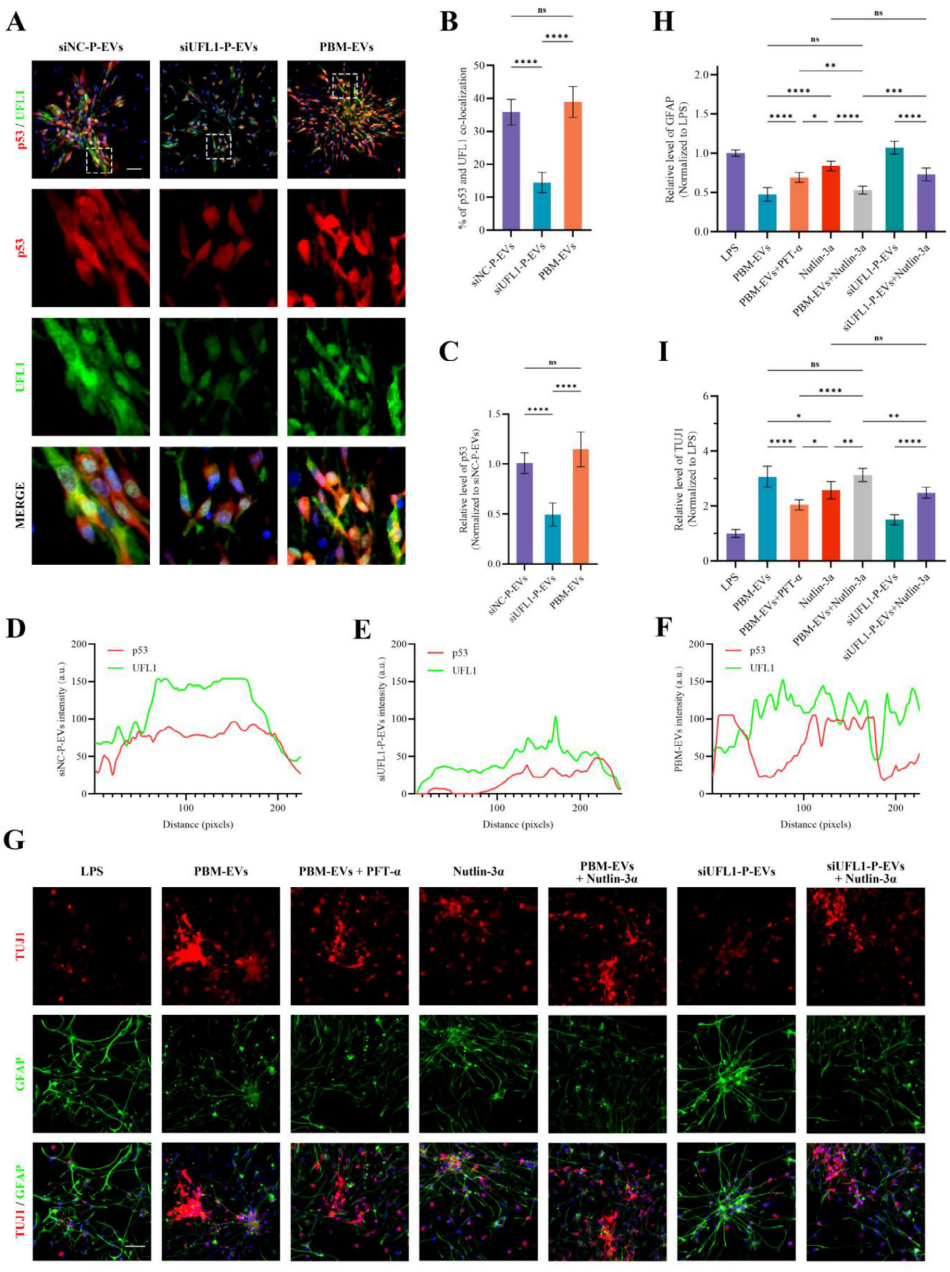
PBM‑EVs activate the p53 signaling pathway by delivering UFL1 to promote neural stem cell differentiation toward neurons. (A, B) Co‑localization of p53 and UFL1 in NSCs. n = 3 independent biological replicates. (A) Representative immunofluorescence images showing p53, UFL1, and DAPI in NSCs (scale bars: 20 µm). (B) Quantitative analysis of p53‑UFL1 co‑localization. (C) Quantitative analysis of p53 protein levels. (D–F) Quantitative analysis of line‑scan fluorescence intensity per single cell in each group. (G–I) Effects of p53 pathway modulation on NSC differentiation under LPS‑induced inflammatory conditions. n = 3 independent biological replicates. (G) Representative immunofluorescence images of Tuj1 and GFAP in NSCs treated with LPS alone, PBM‑EVs, PBM‑EVs + PFT‑α (p53 inhibitor), Nutlin‑3a (p53 stabilizer) alone, PBM‑EVs + Nutlin‑3a, siUFL1‑P‑EVs, or siUFL1‑P‑EVs + Nutlin‑3a (scale bars: 50 µm). (H) Quantification of Tuj1‑positive neurons. (I) Quantification of GFAP‑positive astrocytes. All data are presented as mean ± SD. Statistical analysis: one‐way ANOVA with Tukey's post‐hoc test. ns, *p* > 0.05; ^*^ *p* < 0.05; ^**^ *p* < 0.01; ^***^ *p* < 0.001; ^****^ *p* < 0.0001.

We next determined whether the UFL1/p53 axis is similarly required for the therapeutic efficacy of PBM‐EVs in vivo, using pharmacological inhibition of p53 with PFT‐α (5 mg/kg, i.p., daily for 7 days) in PBM‐EV‐treated rats. As shown in Figure 5, PBM‐EVs significantly improved hindlimb motor recovery at 28 days post‐injury and motor coordination. Co‐administration of PFT‐α significantly abolished these benefits, reducing BBB scores and LSS scores at the corresponding time points, values not significantly different from Control‐EVs (Figure S8A.B). Histologically, PBM‐EVs significantly increased the Tuj1‐positive neuronal area and reduced GFAP‐positive glial scarring compared with Control‐EVs, whereas co‐administration of PFT‐α significantly abolished these effects, yielding Tuj1‐positive and GFAP‐positive areas indistinguishable from Control‐EVs (Figure S8C–E). Similarly, the dense NF‐positive axonal profiles traversing the lesion core in PBM‐EV‐treated animals were obviously absent with PFT‐α co‐treatment (Figure S8F,G). The phenotypic concordance between pharmacological p53 inhibition and genetic UFL1 ablation across behavioral and histological endpoints demonstrates that the UFL1/p53 axis is a principal driver of PBM‐EV therapeutic efficacy in the injured spinal cord.

## 4 Discussion

This study demonstrates that PBM‐EVs significantly promote neural repair after SCI. PBM‐EVs isolated from PBM‐treated microglia were enriched with UFL1 and other functional proteins. In vitro, these EVs protected NSCs from inflammation‐induced apoptosis and directed their differentiation toward a neuronal lineage. In a rat SCI model, PBM‐EVs delivered UFL1 to the injury site, improving the local immune microenvironment, promoting M2 microglial polarization, reducing glial scar formation, and enhancing axon regeneration and hindlimb motor recovery. Mechanistically, UFL1, the only E3 ligase in the UFMylation system, bound p53 competitively with MDM2, thereby inhibiting MDM2‐mediated ubiquitination and degradation of p53, stabilizing p53, and activating the p53 signaling pathway, a key event in UFL1‐regulated neural differentiation (Figure 11).

**FIGURE 11:**
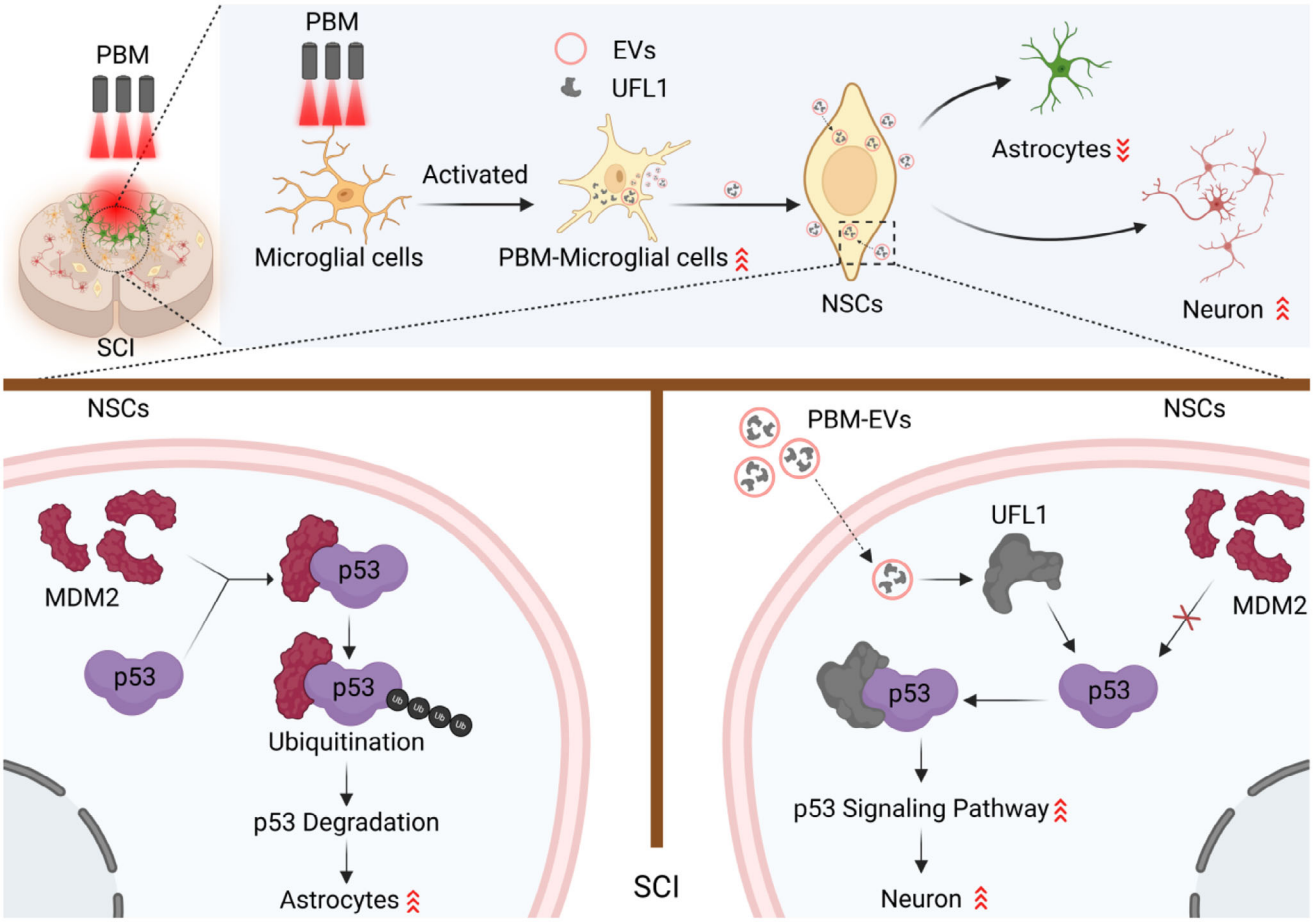
Schematic diagram illustrating the UFL1/p53 axis in PBM‐EVs‐mediated neural differentiation and immune modulation after SCI.

The PBM device used in this study produces biological effects that regulate inflammatory processes and nerve regeneration. The primary mechanism is believed to involve cytochrome c oxidase (CCO), a mitochondrial membrane protein complex that absorbs photons in the 600–1000 nm wavelength range [29]. Photon absorption increases electron flow within the CCO catalytic center, promoting the reduction of molecular oxygen, which leads to elevated mitochondrial membrane potential (MMP), ATP production, and increased levels of cyclic adenosine monophosphate (cAMP) and ROS, thereby enhancing mitochondrial function and initiating downstream signaling [29, 30]. The 850 nm wavelength used here falls within the CCO absorption peak. PBM exhibits Arndt–Schulz biphasic dose responses, according to which low doses activate, and high doses inhibit [31]. Microglia, as central nervous system‐resident immune cells, play a pivotal role in secondary injury and repair after SCI, with their polarization state directly determining the inflammatory response intensity [32, 33]. Thus, inducing anti‐inflammatory M2 polarization is a key therapeutic strategy. Previous studies, including a report by Wang et al., demonstrated that 810 nm PBM inhibited neurotoxic microglia and astrocytes, alleviating neuroinflammation and apoptosis [34]. In the present study, 850 nm PBM at an energy density of 2.0 J/cm^2^ optimally promoted M2 marker expression and suppressed M1 markers. Accordingly, microglia were selected as the PBM target, and their immunomodulatory effects were delivered via EVs, overcoming the limited tissue penetration of PBM by delivering PBM‐induced immunomodulatory signals via EVs. Tail vein injection allowed systemically administered EVs to reach the injury site, facilitated by the increased permeability of the blood‐spinal cord barrier during the acute and subacute phases of SCI. This approach integrates PBM‐induced immunomodulation with EV‐mediated molecular delivery, offering a more systematic intervention than single‐modality strategies.

The pathophysiology of SCI is divided into primary and secondary stages. Primary injury, caused by mechanical force, results in axon rupture, cell necrosis, and disruption of the blood–spinal cord barrier. Secondary injury involves cascade reactions, including inflammatory cell infiltration, oxidative stress, excitotoxicity, and mitochondrial dysfunction, which lead to expansion of the injury area, neuronal and glial cell death, demyelination, and cavity formation [35, 36]. Within this complex pathology, endogenous NSCs (eNSCs) possess proliferation and multi‐lineage differentiation potential, offering a cell source for SCI repair. Numerous studies have confirmed the feasibility of modulating eNSCs for SCI repair. Wang et al. identified key signaling pathways for eNSC differentiation, including Notch, Wnt/β‐catenin, Sonic Hedgehog, and PI3K/Akt [37]. Frisén's group demonstrated that forced OLIG2 expression unlocked the oligodendrocyte differentiation potential in adult spinal cord ependymal stem cells, promoting myelin regeneration and functional recovery [38]. Bai et al. used spinal cord‐derived acellular matrix microgels as a carrier for NSC transplantation, with an in vitro pre‐differentiation strategy that improved the efficiency of neuronal differentiation [39]. In a previous study, we showed that ASIC1A activation prevented the differentiation of NSCs into oligodendrocytes via transcellular PGE2 transmission, impairing myelin regeneration and remyelination [40]. After SCI, eNSCs predominantly differentiate into astrocytes, with only a minority of them becoming neurons, a key factor contributing to failed regeneration. In the present study, PBM‐EV treatment significantly promoted directed differentiation of NSCs into neurons while inhibiting astrocytic differentiation, effectively reversing the differentiation bias within the injury microenvironment. In vivo, the number of neurons in the peri‐injury area was increased, correlating spatially with EV distribution, indicating that PBM‐EVs enhance the regenerative potential of eNSCs by regulating differentiation.

EVs are natural carriers capable of transporting multiple bioactive molecules, enabling synergistic regulatory effects [41]. Engineered EVs have garnered considerable attention in SCI treatment, with studies demonstrating that targeted modification or preconditioning strategies can enhance homing and therapeutic efficacy [42]. Microglia‐derived EVs carry proteins, mRNAs, and miRNAs, mediating intercellular communication that affects neuronal survival, synaptic plasticity, and neuroinflammation [43]. EVs have been shown to reduce disease pathology by delivering neurotrophic factors, restoring synaptic function, or transporting Aβ to microglia for clearance [10, 43, 44]. Microglia‐derived EVs mediate neuronal survival, neurite outgrowth, and neuroinflammatory responses via enzymes, chaperone proteins, membrane receptors, and miRNAs. However, the role and mechanism of PBM‐regulated microglia‐derived EVs in SCI repair remain unclear. PBM‐EVs carried significantly higher levels of UFL1 than M2‐EVs, suggesting that PBM induces a qualitatively distinct M2 polarization state with enhanced UFL1 expression. This effect may occur via mitochondrial retrograde signaling or ROS‐mediated transcriptional reprogramming, and cannot be fully recapitulated by cytokine stimulation alone. This PBM‐specific signature provides a mechanistic rationale for the photobiomodulation approach beyond generic anti‐inflammatory polarization. In this study, EVs isolated from PBM‐treated microglia delivered PBM‐induced M2 polarization effects via vesicle carriers, overcoming the limitations of PBM penetration and enabling broader distribution within the injury site. Proteomic analysis revealed significant differences in protein composition between PBM‐EVs and control EVs. UFL1 was identified as a core effector molecule based on its central position in protein–protein interaction networks and its potential interactions with proteins involved in neural differentiation and immune regulation. This unbiased screening approach avoided the limitations of hypothesis‐driven selection and facilitated the discovery of novel functional molecules. We note several considerations regarding the proteomic nomination of UFL1. First, GO enrichment analysis of PBM‐EV‐associated proteins identified terms including rRNA processing, ribosomal subunit biogenesis, and nucleolar components. While such categories may partly reflect co‐isolation of ribonucleoprotein complexes during differential ultracentrifugation, ribosomal proteins have been consistently identified in high‐confidence EV proteomes across diverse cell types and are increasingly recognized as genuine EV cargo capable of modulating translation in recipient cells. The presence of established EV markers (CD9, CD63, TSG101) and the absence of the ER marker Calnexin support that our preparations are enriched in bona fide small EVs, though orthogonal purification methods such as iodixanol density gradient flotation would further resolve the contribution of co‐isolated non‐vesicular proteins in future studies. Second, while UFL1 knockdown abrogated PBM‐EV therapeutic effects, formally establishing UFL1 as necessary, we did not test whether UFL1 alone is sufficient to recapitulate the full PBM‐EV phenotype. Given that 445 proteins were significantly upregulated in PBM‐EVs, additional cargo molecules likely cooperate with UFL1. Notably, proteins enriched in ECM–receptor interaction and chromatin remodeling pathways may contribute to the adhesion remodeling and transcriptional reprogramming observed in recipient NSCs. Future gain‐of‐function studies using UFL1‐overexpressing donor microglia or recombinant UFL1‐loaded EVs will be required to dissect the individual and combinatorial contributions of PBM‐EV cargo components.

UFMylation is a recently identified ubiquitin‐like modification involved in diverse biological processes, including maintenance of endoplasmic reticulum homeostasis, cell differentiation, DNA damage response, and cancer‐related signaling pathways. It requires a three‐step enzymatic cascade involving E1 (UBA5), E2 (UFC1), and E3 (UFL1) [23, 45]. UFL1, the only E3 ligase in this system, has been increasingly recognized for its pathophysiological roles. Cardiac‐specific UFL1 knockout leads to perinatal cardiomyopathy, attributed to excessive endoplasmic reticulum stress, impaired mitochondrial oxidative metabolism, and sustained mTOR signaling [46]. Growth factor‐induced AKT phosphorylation of UFL1 at threonine 426 promotes UFMylation of ArpC4, activating the Arp2/3 complex to drive pseudopodia formation and accelerate tumor metastasis [47]. These studies highlight the functional diversity of UFL1‐mediated UFMylation across tissues and cell types. In the present study, UFL1 was linked to neural differentiation and immune regulation, expanding the known functions of the UFMylation system. Notably, the abundance of UFL1 was significantly increased in PBM‐EVs, and UFL1 knockdown nearly abolished the therapeutic effects, indicating that UFL1 is not merely a marker but a core functional mediator. Unlike strategies using conventional engineered EVs that rely on literature‐based selection of neurotrophic factors or miRNAs, in this study, we employed a proteomics approach to identify functional molecules from donor cell responses, offering a strategy for discovering novel effectors and laying a foundation for mechanistic studies.

The involvement of p53 signaling was an intriguing finding with emerging mechanistic support. While p53 is best characterized as a tumor suppressor that promotes apoptosis, cell‐cycle arrest, and senescence, a growing body of evidence indicates context‐dependent roles in stem cell biology [48, 49]. Recent studies have shown that UFMylation stabilizes p53 by antagonizing its ubiquitination, identifying p53 as a UFMylation substrate of UFL1 [25, 50]. As a core transcription factor, the protein levels of p53 directly affect the expression of downstream genes involved in neural differentiation, acting as a molecular switch that determines the fate of NSCs toward neurons or astrocytes. Proteasome activity dynamically changes during NSC differentiation, regulating neurogenesis via stabilization of key transcription factors [51, 52]. We confirmed a physical interaction between UFL1 and p53, with UFL1 knockdown shortening the half‐life of p53 and increasing its ubiquitination. UFL1 competitively binds p53 with MDM2, inhibiting MDM2‐mediated ubiquitination and degradation. p53 regulates hundreds of genes involved in cell growth, division, survival, death, and stress responses [53, 54, 55]. Under homeostatic conditions, p53 is short‐lived; under stress, it is stabilized via post‐translational mechanisms, promoting growth arrest, senescence, or apoptosis [56, 57]. In the central nervous system, p53 regulates the self‐renewal and differentiation of NSCs independently of its canonical apoptotic functions. Turnquist et al. demonstrated that p53‐null NSCs exhibit enhanced proliferation and preferential astrocytic differentiation, suggesting that p53 actively promotes neuronal lineage commitment [58]. However, its specific role in NSC differentiation after SCI remained unclear, a gap that we addressed in this study. We found that UFL1 delivery via PBM‐EVs upregulated p53 and its downstream targets in NSCs. p53 inhibition completely blocked PBM‐EV‐induced neuronal differentiation, and time kinetics showed that the increase in p53 levels preceded the expression of neuronal markers, confirming p53 as an upstream regulator. In NSCs treated with UFL1‐knockdown EVs, p53 levels were unchanged and the pro‐differentiation effect was lost, but exogenous p53 partially rescued this effect, confirming that UFL1 regulates NSC differentiation through the p53 pathway. A complex interplay exists between neuroinflammation and p53 signaling. Nuclear factor κB (NF‐κB), a core pro‐inflammatory transcription factor, activates the NLRP3 inflammasome, promoting IL‐1β release and exacerbating inflammation [59]. p53 can inhibit NF‐κB activity by competing for transcriptional coactivators or inducing IκBα expression [60]. In this study, PBM‐EV treatment promoted M2 microglial polarization and reduced the levels of pro‐inflammatory cytokines, effects that may be partly mediated by p53‐dependent NF‐κB inhibition, suggesting that the p53 pathway contributes to UFL1‐mediated regulation of the immune microenvironment. However, SCI repair involves multiple cell types, including neurons, astrocytes, and oligodendrocytes, and the effects of UFL1 in PBM‐EVs on these cells warrant further investigation.

## 5 Conclusion

In summary, this study is the first to establish and validate the “PBM–EV–UFL1–p53” axis as a synergistic therapeutic strategy for SCI. PBM‐EVs activate the p53 signaling pathway through UFL1 delivery, exerting combined effects, including immune regulation, neuroprotection, and promotion of neural differentiation, ultimately enhancing functional recovery. The mechanistic insights obtained in this study suggest that leveraging endogenous cellular responses may be more effective than introducing exogenous factors. Furthermore, this study offers a new perspective on the interplay between protein modification networks, where competition between ubiquitination (which typically marks proteins for degradation) and UFMylation (which stabilizes p53) determines the steady‐state levels of key regulatory proteins.

## Author Contributions

C.S. and L.C. contributed to the conception and design of the work. Y.F. contributed to the research design, acquisition, and analysis of the data, and manuscript writing. Z.W. and R.G. contributed to the manuscript writing, analysis, and interpretation of the data. Y.F., Y.D., C.W., C.J., Y.Q., A.L., and A.L. contributed to the acquisition of the data and software used in the work. T.H. and P.S. contributed to the acquisition and analysis of the data. All the authors have read and approved the final manuscript.

## Funding

This work was supported by the National Natural Science Foundation of China project (No. 82572752), Anhui Provincial Key R&D Program project (2022e07020046), and Anhui Medical University Research Fund project (No. 2023xkj088).

## Ethics Statement

SD rats were provided by the Animal Experiment Centre of Anhui Medical University. All experimental procedures involving animals were strictly conducted in accordance with the NIH guidelines for the care and use of laboratory animals and were approved by the Ethics Committee of Anhui Medical University (Approval No.: LLSC20242471).

## Conflicts of Interest

The authors declare no conflicts of interest.

## Supporting information




**Supporting File 1**: advs77237‐sup‐0001‐SuppMat.docx.

**Supporting File 2**: advs77237‐sup‐0002‐TableS1.docx.

## Data Availability

The data that support the findings of this study are available from the corresponding author upon reasonable request.
