# Supplementary material for: Photobiomodulation‑Engineered Extracellular Vesicles Enhance Neural Differentiation via UFL1‑Mediated UFMylation in Spinal Cord Injury
Source: Adv Sci (Weinh). 2026 Aug 18;13(62):e77237. doi: 10.1002/advs.77237 (PMC13483136; doi:10.1002/advs.77237)
Supplement: Supplementary file 2 [file ADVS-13-e77237-s002.docx]

Supplementary Fig. 1 (A, B) TUNEL staining of spinal cord sections on day 14 post‑injury. n = 5 animals per group for histological analyses. (A) Representative images of TUNEL and DAPI in the peri‑injury region from sham, SCI,  Control‑EVs, and PBM‑EVs groups (scale bars, 50 μm). (B) Quantification of TUNEL‑positive cells. (C, D) Nissl staining of spinal cord sections on day 14 post‑injury. (C) Representative images showing Nissl bodies (purple) in neurons at the injury center (scale bars, 100 μm). (D) Quantification of surviving neurons per section. All data are representative of at least three independent experiments. n = 5 animals per group for histological analyses. All data are presented as mean ± SD. Statistical analysis: one-way ANOVA with Tukey's post-hoc test. ns, P > 0.05; *P < 0.05; **P < 0.01; ***P < 0.001; ****P < 0.0001.

Supplementary Fig. 2 (A) Representative HE‑stained spinal cord sections showing the injury cavity on day 28 post‑injury for cavity area analysis (scale bars, 200 μm). (B) Quantification of cavity area. (C) Representative images of bladder cross‑sections on day 28 for bladder structure analysis. (scale bars, 1 mm). (D) Quantification of bladder wall thickness. (E) Quantification of bladder weight. (F) Safety evaluation: HE staining of heart, liver, spleen, lung, and kidney from sham, SCI, and PBM‑EV‑treated rats (scale bars, 200 μm). No obvious structural abnormalities or toxicity were observed in any organ. n = 5 per group for cavity and bladder analyses; n = 3 per group for organ safety evaluation. All data are presented as mean ± SD. Statistical analysis: one-way ANOVA with Tukey's post-hoc test. ns, P > 0.05; *P < 0.05; **P < 0.01; ***P < 0.001; ****P < 0.0001.

Supplementary Fig. 3 (A–E) ELISA measurements of pro‑inflammatory cytokines TNF‑α (A), IL‑1β (B), IL‑6 (C) and anti‑inflammatory cytokines IL‑4 (D), IL‑10 (E) in NSC culture supernatants. All data are representative of at least three independent experiments. n = 3 independent experiments, each assayed in duplicate. All data are presented as mean ± SD. Statistical analysis: one-way ANOVA with Tukey's post-hoc test. ns, P > 0.05; *P < 0.05; **P < 0.01; ***P < 0.001; ****P < 0.0001.

Supplementary Fig. 4 (A) qRT‑PCR validation of UFL1 mRNA levels in independent batches of EVs. n = 3 independent biological replicates, each assayed in triplicate. (B) Western blot analysis of independent EV preparations, showing that UFL1 protein abundance was significantly elevated in PBM‑EVs relative to Control‑EVs, corroborating the proteomic and qRT‑PCR results. n = 3 independent experiments; representative blot shown. Densitometry normalized to loading control. (C) qRT‑PCR confirmation of UFL1 knockdown efficiency in donor microglia after siRNA transfection. (D–H) ELISA analysis of pro‑inflammatory (TNF‑α, IL‑1β, IL‑6) and anti‑inflammatory (IL‑4, IL‑10) cytokines in microglia culture supernatants after treatment with different EVs. n = 3 independent experiments, each assayed in duplicate. All data are presented as mean ± SD. Statistical analysis: one-way ANOVA with Tukey's post-hoc test. ns, P > 0.05; *P < 0.05; **P < 0.01; ***P < 0.001; ****P < 0.0001.

Supplementary Fig. 5 (A) Representative triple immunofluorescence images of iNOS, Arg1, Iba1, and DAPI in spinal cord sections at the injury site on day 7 post-injury from rats treated with siNC-P-EVs, siUFL1-P-EVs, or PBM-EVs (Scale bars, 50 μm). (B) Quantification of Arg1⁺/Iba1⁺ cell proportion. (C) Quantification of iNOS⁺/Iba1⁺ cell proportion. n = 5 animals per group for histological analyses. All data are presented as mean ± SD. Statistical analysis: one-way ANOVA with Tukey's post-hoc test. ns, P > 0.05; *P < 0.05; **P < 0.01; ***P < 0.001; ****P < 0.0001.

Supplementary Fig. 6 (A, B) Representative images of Arg1/Iba1 and iNOS/Iba1 co‑localization (scale bars, 50 μm). n = 3 independent biological replicates. (C, D) corresponding quantitative analyses. (E) Western blot analysis of UFL1 protein in EVs derived from PBM‑EVs versus M2‑EVs. n = 3 independent experiments; representative blot shown. Densitometry normalized to loading control. All data are presented as mean ± SD. Statistical analysis: one-way ANOVA with Tukey's post-hoc test. ns, P > 0.05; *P < 0.05; **P < 0.01; ***P < 0.001; ****P < 0.0001.

Supplementary Fig. 7 (A) Representative immunofluorescence images of EdU and NeuN co‑localization in spinal cord sections (scale bars, 50 μm). (B) Quantification of neurons double‑positive for EdU and NeuN. (C) BBB scores over 4 weeks post‑injury. (D) LSS scores over 4 weeks post‑injury, showing similar trends. (E) Gene set enrichment analysis (GSEA) showing significant enrichment of p53 signaling pathway‑related genes in PBM‑EV‑treated samples compared with controls. n = 5 animals per group for histological analyses; n = 8 for behavioral data. All data are presented as mean ± SD. Statistical analysis: one-way ANOVA with Tukey's post-hoc test. ns, P > 0.05; *P < 0.05; **P < 0.01; ***P < 0.001; ****P < 0.0001.

Supplementary Fig. 8 (A) Basso‑Beattie‑Bresnahan (BBB) locomotor rating scale scores. (B) LSS assessments. (C) Representative images of Tuj1 and GFAP staining in spinal cord sections (scale bars, 200 μm). (D) Quantification of Tuj1‑positive neuronal area. (E) Quantification of GFAP‑positive glial scar area. (F) Representative images of NF and GFAP co‑staining in sections of the injured spinal cord (scale bars, 200 μm). (G) Quantification of NF‑positive fluorescence intensity across the injury site presented as a line graph. n = 5 animals per group for histological analyses; n = 8 for behavioral data. All data are presented as mean ± SD. Statistical analysis: one-way ANOVA with Tukey's post-hoc test. ns, P > 0.05; *P < 0.05; **P < 0.01; ***P < 0.001; ****P < 0.0001.

**ARRIVE 2.0 Guidelines Checklist**

Essential 10

| **Item** | **Location in Manuscript** |
| --- | --- |
| **1. Study design** | (a) §2.6: Six groups described—Sham, SCI + PBS, SCI + Control-EVs, SCI + PBM-EVs, SCI + siUFL1-P-EVs, SCI + PBM-EVs + PFT-α.  (b) §2.21: Individual animal is the experimental unit. For in vitro experiments, independent biological replicates (separate cell cultures on different days) as defined in §2.21. |
| **2. Sample size** | (a) §2.6: n = 8 per group for behavioral assessments; n = 5 per group for histological/biochemical analyses. Total enrolled: sham n = 8; injury groups n = 10 each (58 total). Supplementary Table S1: complete animal flow. All figure legends: specific n values per experiment type.  (b) §2.21: A priori power analysis using G*Power 3.1.9.7 (effect size f = 0.65, α = 0.05, power = 0.80, one-way ANOVA, 6 groups) based on pilot BBB score data. |
| **3. Inclusion and exclusion criteria** | (a) §2.21: Pre-specified exclusion criteria: (i) death within 72 h of surgery; (ii) autophagia or self-mutilation requiring early euthanasia; (iii) incomplete injury defined as BBB score > 3 at day 1. No animals excluded after day 7.  (b) Supplementary Table S1: exclusions by category per group.  (c) All figure legends: exact n per group per experiment. §2.21: n = 8 (behavioral); n = 5 (histological/biochemical). |
| **4. Randomisation** | (a) §2.6: Computer-generated block randomization sequence (block size = 3; GraphPad QuickCalcs). Allocation concealed in sequentially numbered opaque envelopes opened after SCI induction. §2.2: In vitro PBM irradiation order randomized using random number table, re-randomized per independent experiment. §2.10: Slide identity masked; imaging order randomized across groups.  (b) §2.21: Independent investigator generated allocation; surgeon blinded to allocation; treatment sequence re-randomized per experiment; slide identities masked and imaging order randomized. |
| **5. Blinding** | §2.21: (i) Surgeon blinded during SCI induction; (ii) Two independent observers blinded for all behavioral assessments (§2.8); (iii) Histological quantification (§2.10), Western blot densitometry (§2.17), and flow cytometry gating performed with group identities masked by numerical codes; (iv) Statistical analysis performed on de-identified datasets. Codes revealed only after all analyses complete. |
| **6. Outcome measures** | (a) §2.8: BBB open-field test, inclined plane test, LSS swimming test, footprint analysis (stride length, toe spread). §2.9: MEP amplitude. §2.10: Immunofluorescence quantification (iNOS⁺/Iba1⁺, Arg1⁺/Iba1⁺, Tuj1⁺ area, GFAP⁺ area, NeuN⁺, NF⁺ intensity). §2.11: HE staining (cavity area), Nissl staining. §2.12: CCK-8 viability. §2.13: TUNEL and flow cytometry apoptosis. §2.14: ROS (DCFH-DA, DHE). §2.15: qRT-PCR. §2.16: ELISA (TNF-α, IL-1β, IL-6, IL-4, IL-10). §2.17: Western blot.  (b) §2.21: Primary outcome for sample size determination was 28-day BBB score (effect size derived from observed 3.7-point difference between PBM-EV and Control-EV groups in pilot study). |
| **7. Statistical methods** | (a) §2.21: GraphPad Prism 8 and SPSS 26.0. Two-group comparisons: unpaired two-tailed Student's t-test. Multiple-group comparisons: one-way ANOVA with Tukey's HSD post-hoc. Longitudinal behavioral data: repeated-measures two-way ANOVA (group × time) with Geisser-Greenhouse correction. Non-parametric: Mann-Whitney U test or Kruskal-Wallis with Dunn's correction.  (b) §2.21: Shapiro-Wilk test for normality. Non-parametric alternatives applied when normality rejected. Geisser-Greenhouse correction applied when Mauchly's test indicated violation of sphericity. |
| **8. Experimental animals** | (a) §2.6: Female Sprague-Dawley (SD) rats, 8–10 weeks old, approximately 220 g. §2.1: Primary microglia isolated from cerebral cortex of newborn rats. §2.5: Primary NSCs from E14–E16 fetal rats.  (b) §2.6: Obtained from Laboratory Animal Center of Anhui Medical University (Hefei, China). Housed under SPF conditions, 22–24°C, 40–60% humidity, 12 h light/dark cycle, ad libitum food/water. No genetic modifications. No previous procedures. Female rats selected to avoid aggressive behavior confounds after SCI (§2.6). |
| **9. Experimental procedures** | (a–c) §2.6: SCI induced at T9–T10 by complete bilateral compression (Dumont microforceps, tip width 0.5 mm, 5 s) under pentobarbital anesthesia (35 mg/kg, i.p.). EVs (100 μg in 200 μL PBS) via tail vein injection on day 0, 1, 3, 7. PFT-α (5 mg/kg i.p. in 10% DMSO/saline) daily × 7 days. Postoperative antibiotics twice daily; manual bladder expression. §2.8: Behavioral assessments on days 1, 3, 7, 14, 21, 28. §2.7: In vitro PFT-α (10 μM) and Nutlin-3a (5 μM) with 2 h pre-incubation.  (d) §2.6: Compression model selected for clinical relevance; tail vein route selected for systemic EV delivery; female rats selected for injury uniformity and animal welfare. |
| **10. Results** | (a) All Results sections (§3.1–§3.11): Summary statistics reported as mean ± SD throughout. All figure legends: mean ± SD with exact n values. Figure panels display individual data points or bar graphs with error bars.  (b) §2.21: Effect size f = 0.65 reported for sample size calculation. §3.5: BBB scores at 28 days (PBM-EVs: 10.5 ± 1.2 vs Control-EVs: 6.8 ± 0.9). §3.11: PFT-α group BBB scores (7.2 ± 1.0) reported alongside comparator groups. Full statistical reporting with exact P values in figures. |

Supplementary Table S1: Animal Flowchart

| **Group** | **Enrolled (n)** | **Excluded: Death < 72 h (n)** | | **Excluded: Autophagia (n)** | **Excluded: BBB > 3 at day 1 (n)** | **Included in Final Analysis (n)** |
| --- | --- | --- | --- | --- | --- | --- |
| Sham | 8 | 0 | 0 | | 0 | 8 |
| SCI + PBS | 10 | 1 | 0 | | 1 | 8 |
| SCI + Control-EVs | 10 | 0 | 1 | | 1 | 8 |
| SCI + PBM-EVs | 10 | 1 | 0 | | 1 | 8 |
| SCI + PBM-EVs + PFT-α | 10 | 1 | 0 | | 1 | 8 |
| SCI + siUFL1-P-EVs | 10 | 1 | 0 | | 1 | 8 |
