# Supplementary material for: Photobiomodulation‑Engineered Extracellular Vesicles Enhance Neural Differentiation via UFL1‑Mediated UFMylation in Spinal Cord Injury
Source: Adv Sci (Weinh). 2026 Aug 18:e77237. Online ahead of print. doi: 10.1002/advs.77237 (PMC13483136; doi:10.1002/advs.77237)
Supplement: Supplementary file 2 [file ADVS-9999-e77237-s002.docx]

Supplementary Table S1: Animal Flowchart

| **Group** | **Enrolled (n)** | **Excluded: Death < 72 h (n)** | | **Excluded: Autophagia (n)** | **Excluded: BBB > 3 at day 1 (n)** | **Included in Final Analysis (n)** |
| --- | --- | --- | --- | --- | --- | --- |
| Sham | 8 | 0 | 0 | | 0 | 8 |
| SCI + PBS | 10 | 1 | 0 | | 1 | 8 |
| SCI + Control-EVs | 10 | 0 | 1 | | 1 | 8 |
| SCI + PBM-EVs | 10 | 1 | 0 | | 1 | 8 |
| SCI + PBM-EVs + PFT-α | 10 | 1 | 0 | | 1 | 8 |
| SCI + siUFL1-P-EVs | 10 | 1 | 0 | | 1 | 8 |
